# Lacritin proteoforms prevent tear film collapse and maintain epithelial homeostasis

**DOI:** 10.1074/jbc.RA120.015833

**Published:** 2020-11-21

**Authors:** Georgi A. Georgiev, Mohammad Sharifian Gh., Jeff Romano, Karina L. Dias Teixeira, Craig Struble, Denise S. Ryan, Rose K. Sia, Jay P. Kitt, Joel M. Harris, Ku-Lung Hsu, Adam Libby, Marc G. Odrich, Tatiana Suárez, Robert L. McKown, Gordon W. Laurie

**Affiliations:** 1 Institute for Bioengineering and Biosciences, Instituto Superior Técnico, Universidade de Lisboa, Lisbon, Portugal; 2 Department of Cell Biology, University of Virginia, Charlottesville, Virginia, USA; 3 Drug Metabolism, Covance Laboratories Inc, Madison, Wisconsin, USA; 4 Warfighter Refractive Eye Surgery Program and Research Center at Fort Belvoir, Fort Belvoir, Virginia, USA; 5 Department of Chemistry, University of Utah, Salt Lake City, Utah, USA; 6 Department of Chemistry, University of Virginia, Charlottesville, Virginia, USA; 7 Department of Ophthalmology, University of Virginia, Charlottesville, Virginia, USA; 8 Department of Research, Development and Innovation, FAES FARMA, Bizkaia, Spain; 9 Department of Integrated Science and Technology, James Madison University, Harrisonburg, Virginia, USA; 10 Department of Biomedical Engineering, University of Virginia, Charlottesville, Virginia, USA

**Keywords:** lacritin, proteoform, eye, tears, OAHFA, protein–lipid interaction, elastic modulus, Raman spectroscopy, proteolysis, epithelium, AAAS, aladin WD repeat nucleoporin, ABCA3, ATP binding cassette subfamily A member 3, CLDN10, claudin 10, Cyp4f39, cytochrome P450, family 4, subfamily f, polypeptide 39, E_R_, stored elastic modulus, E_IM_, loss modulus, EMC3, ER membrane protein complex subunit 3, FGF10, fibroblast growth factor 10, FGFR2, fibroblast growth factor receptor 2, FGFR3, fibroblast growth factor receptor 3, FOXC2, forkhead box C2, FWHM, full width at half maximum, HCE-T, human corneal epithelial, NGLY1, N-glycanase 1, OAHFA, (O-acyl)-ω-hydroxy fatty acid, SFTPB, surfactant protein B, SFTPC, surfactant protein C, TGFBI, transforming growth factor beta induced, TP63, tumor protein p63, TRAPPC11, trafficking protein particle complex 11

## Abstract

Lipids in complex, protein-enriched films at air/liquid interfaces reduce surface tension. In the absence of this benefit, the light refracting and immunoprotective tear film on eyes would collapse. Premature collapse, coupled with chronic inflammation compromising visual acuity, is a hallmark of dry eye disease affecting 7 to 10% of individuals worldwide. Although collapse seems independent of mutation (unlike newborn lung alveoli), selective proteome and possible lipidome changes have been noted. These include elevated tissue transglutaminase and consequent inactivation through C-terminal cross-linking of the tear mitogen lacritin, leading to significant loss of lacritin monomer. Lacritin monomer restores homeostasis *via* autophagy and mitochondrial fusion and promotes basal tearing. Here, we discover that lacritin monomer C-terminal processing, inclusive of cysteine, serine, and metalloproteinase activity, generates cationic amphipathic α-helical proteoforms. Such proteoforms (using synthetic peptide surrogates) act like alveolar surfactant proteins to rapidly bind and stabilize the tear lipid layer. Immunodepletion of C- but not N-terminal proteoforms nor intact lacritin, from normal human tears promotes loss of stability akin to human dry eye tears. Stability of these and dry eye tears is rescuable with C- but not N-terminal proteoforms. Repeated topical application in rabbits reveals a proteoform turnover time of 7 to 33 h with gradual loss from human tear lipid that retains bioactivity without further processing. Thus, the processed C-terminus of lacritin that is deficient or absent in dry eye tears appears to play a key role in preventing tear film collapse and as a natural slow release mechanism that restores epithelial homeostasis.

At air/liquid interfaces, lipids in complex protein-enriched films reduce surface tension without which lung alveoli would collapse, as observed in newborns with mutations in pulmonary surfactant genes (*i.e.*, SFTPB; (1), SFTPC; (2)) or in proteins involved in phosphatidylglycerol and phosphatidylcholine (ABCA3; (3)) or protein transport (EMC3; (4)). In the terrestial vertebrate eye, the air/liquid interface is responsible for refracting 80% of entering light, premature collapse of which underlies the most common eye disease (“dry eye”) affecting 7 to 10% of the world's population increasing to 30% in the elderly (5). No single gene mutation nor group of mutations are known to be causative, although in humans, rare frameshift mutation of the transcription factor FOXC2 (6) or R124H mutation of collagen binding TGFBI (7) is deleterious for morphogenesis of eyelid meibomian glands, as are rare mutations of AAAS (8), CLDN10 (9), FGF10, FGFR2, FGFR3 (10, 11), NGLY1 (12), TP63 (13), or TRAPPC11 (14) for formation of the ocular lacrimal glands. How the lipid film of the eye is stabilized is an important question with considerable physiological and health relevance.

Clues may reside in tear lipidomic and proteomic analyses and perhaps *via* singular features shared with lung alveoli. Only the (O-acyl)-ω-hydroxy fatty acid (OAHFA) class of amphiphilic lipids appears to be downregulated in dry eye (15), much like phosphatidylcholine deficiency in infant respiratory distress syndrome (16), and in keeping with “evaporative” dry-eye-like conditions in mouse eyes lacking the Cyp4f39 gene coding for a fatty acid ω-hydroxylase necessary for the generation of normal levels of C16:1 OAHFA (17). OAHFA lipids are thought to primarily reside at the aqueous/lipid interface as the main lipid surfactant essential for tear film stability (18). Also, in dry eye, surprisingly few tear proteins are selectively deficient. Of tear deficient proteins curiously also constituents of broncheoalveolar lung lavage (19, 20) are: zymogen granule protein 16B, annexin A5, alpha-2-glycoprotein 1, deleted in malignant brain tumors1, lipocalin-1, submaxillary gland androgen regulator protein 3B, immunoglobulin heavy constant alpha 1, polymeric immmunoglobulin and lacritin. Lacritin is a basal tearing agonist (21, 22, 23, 24) that in addition transiently stimulates autophagy in ocular surface epithelia (25) to restore oxidative phosphorylation under conditions of inflammatory stress (25). Rather than known mutational inactivation, lacritin is subject to tissue transglutaminase-dependent cross-linking involving donor lysines 82 and 85 and acceptor glutamine 106 (26)—the latter residing within the binding domain of lacritin coreceptor syndecan-1. This abrogates binding and in turn lacritin activity (26). Tissue transglutaminase is elevated both in dry eye (27) and in disrupted alveoli of premature infants with bronchopulmonary dysplasia (28) and through cross-linking diminishes bioactive monomeric lacritin in place of inactive polymers (26).

A further curiosity is lacritin's robust C-terminal processing in normal tears (29, 30) that in proteoform number exceeds that of all but one other tear protein (30). Some are bactericidal (29, 30). Here we note that C-terminal proteoforms are also deficient or absent in dry eye and that this deficit goes hand in hand with the propensity for tear film collapse in a C-terminal synthetic peptide rescuable manner involving stable interaction with 16:1 OAHFA.

## Results

### Lacritin immunodepleted normal tears prematurely collapse

**Figure 1:**
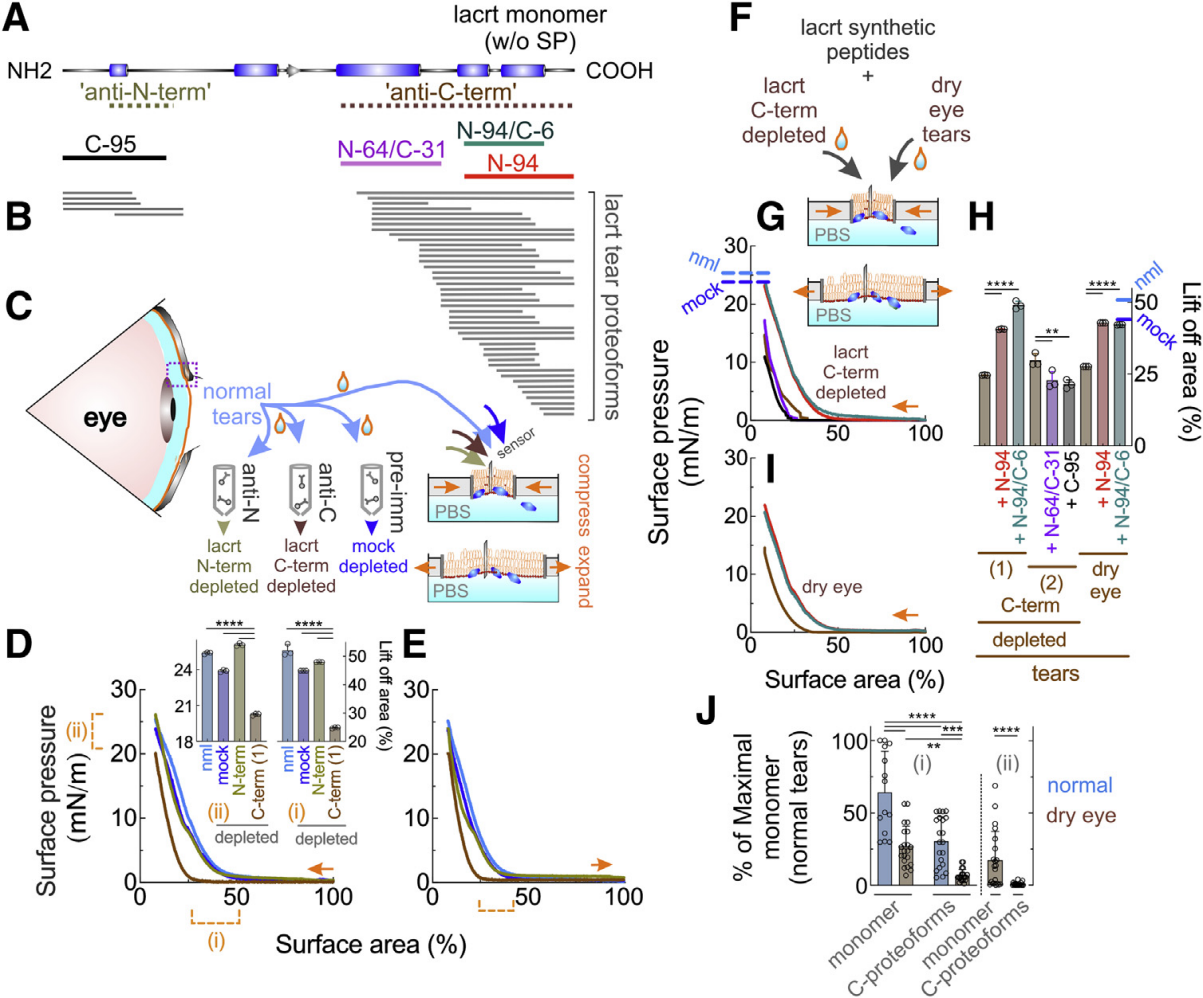
**Lacritin C-terminal proteoforms in normal human tears are essential for tear film stability.***A*, linear diagram of secreted lacritin (lacrt) monomer with rectangles indicative of demonstrated (C-terminal half; (29, 61)) or PSIPRED (v4.0) predicted (N-terminal half) α-helices or beta strand (*arrow head*); *dotted lines* representing respective antigens of monoclonal “anti-N-term” antibody “1F5” and polyclonal “anti-C-term” antibody (26); and alignment of lacritin synthetic peptides “C-95,” “N-64/C-31,” “N-94,” and “N-94/C-6.” *B*, lacritin N- and C-terminal proteoforms detected in normal human tears by top-down mass spectrometry ((30); used with permission). *C*, schematic diagram of lipid (*orange*) and aqueous (*aqua*) portions of normal tears on the eye, and pooled collections of normal human basal tears from 50 different individuals for Langmuir trough compression/expansion isocyling (schematic diagrams at right) without or after immunodepletion over immobilized “anti-N” term, “anti-C” term lacritin antibodies or preimmune (pre-imm) Ig. *Dashed box* on the eye indicates the region highlighted in Figure 3*A*. *D*, tear films (3 μl) on a 80 ml PBS subphase were equilibrated for 15 min under cover and then subjected to compression and expansion isocycling by respectively advancing or retreating dual opposing barriers at 1.37 cm^2^ per second with surface pressure monitored by a Wilhelmy wire probe with sensitivity exceeding 0.01 mN/m (44). In this and subsequent studies, each isotherm represents the mean of triplicate experiments with individual experiments representing over 1200 data points. (i) indicates the “lift off area” at which compression elevates surface pressure from baseline to at least 1 mN/m. (ii) indicates the highest surface pressure attained. *Inset*, comparative highest surface pressure attained (*left*) and lift-off area (*right*) (mean with S.D [n = 3], ∗∗∗∗*p* < 0.0001 [one-way ANOVA with Tukey multiple comparisons test]). *E*, expansion isotherm of the same samples, with bracket highlighting touch-down areas (n = 3). *F*, schematic diagram of compression and expansion isocycling of anti-C-term lacritin-depleted tear films or human aqueous deficient dry eye basal tears (pooled collections each from 50 different individuals) without or supplemented with 6 μM lacritin synthetic peptides C-95, N-64/C-31, N-94, or N-94/C-6. *G*, compression isotherms of anti-C-term lacritin-depleted tear films without or with each peptide per color code in *A* and *H*. *H*, comparative lift-off area (mean with S.D [n = 3], ∗∗∗∗*p* < 0.0001 [one-way ANOVA with Tukey multiple comparisons test]). (1) and (2) are two different anti-C-terminal lacritin-immunodepletions. *I*, expansion isotherm of dry eye tear films without or with N-94 or N-94/C-6 (n = 3). *J,* LI-COR Western blot analysis of relative levels of lacritin monomer and C-terminal proteoforms in individual basal tears from (i) 21 normal (shown are monomer and proteoform data respectively from 14 and 21 individuals), or 21 aqueous deficient dry eye individuals, and tears collected without anesthesia from (ii) 21 Secondary Sjögren's syndrome dry eye patients. Values were normalized to equal protein ([mean with S.D], ∗∗∗∗*p* < 0.0001; ∗∗∗*p* = 0.0001; ∗∗*p* = 0.0013 [one-way ANOVA with Tukey multiple comparisons test]). Data for *D–E*, *G*–*J* in Data files Figure S1.

Tears comprise a loose aqueous polymer of lipids and glycoproteins, including the basal tearing (21, 22, 23, 24) and prohealth (25, 31) agonist lacritin whose bioactive C-terminus is dominated by two amphipathic α-helices (Fig. 1, *A*–*B*) similar to the N- and C-termini of processed lipid stabilizing pulmonary surfactant protein B (32, 33, 34) necessary for lung function and life (35). Together, both lacritin α-helices partially comprise the antigen of polyclonal antibody “anti-C-term” (Fig. 1*A*; (26)), the latter effective for lacritin immunodepletion (25, 26, 29). To gain insight into premature tear film collapse and whether lacritin deficiency in dry eye may be contributory, Langmuir surface balance studies were performed. Such studies are performed in a “Langmuir-Blodgett trough” consisting of a shallow (225 cm^2^) reservoir of PBS (40 ml) with surface tear film that is compressed or expanded by symmetric movement of opposing barriers. As area changes, surface pressure is monitored by a Wilhelmy wire probe with sensitivity exceeding 0.01 mN/m. Surface pressure represents the surface tension of the buffer minus the surface tension of the film. We floated onto PBS normal human tears (3 μl) pooled from over 50 different individuals, or the same tear pools passed over immobilized anti-C-term lacritin antibodies (*C-term depleted*; Fig. S1*A*), or over a preimmune Ig column (Fig. 1*C*; *mock depleted*). Each was subjected to compression and expansion isocycling (Fig. 1*C*, *right*) that mimics blinking. Isocycling is the synchronized repeated inward or outward movement of barriers at constant speed. Isocycling was performed at 35 °C, the surface temperature of the human eye (36). Normal and mock depleted films (Fig. 1, *D*–*E*) were more stable as per a higher “lift off” area (∼50%; Fig. 1*D*(i), *D inset*, *right*) and maximum surface pressure (∼25 mN/m; Fig. 1*D*(ii), *D inset*, *left*) achieved with compression. “Lift off” is when surface pressure first reaches 1 mN/m as compression progressively reduces film area (37)—an indicator of the capability of film constituents to restructure at the interface. Maximum surface pressure is when constituents are the most densely compressed in a stable film. Tears lacking lacritin were less resistant to compression (respectively ∼25% and ∼15–20 mN/m; Fig. 1*D* (i and ii), *D inset*, *right and left*) and expansion (Fig. 1*E*). Film stability is considered in terms of molecular packing density at the interface (38).

### Anti-C-term lacritin depleted tears behave like dry eye tears and can be rescued with C-Terminal lacritin peptides that are deficient in dry eye

Normal basal tears contain different forms of lacritin as monomer, polymer, and proteoforms (26) - of which five N- and 42 different C-terminal proteoforms have been detected to date by top-down mass spectrometry ((30); Fig. 1*B*: “lacrt tear proteoforms”). Lacritin dimers, trimers, and larger polymers are attributable to cross-linking by tear tissue transglutaminase, a calcium-dependent glutamine γ-glutamyltransferase that is active in normal tears (26) and whose ocular surface expression is elevated in patients with Sjögren's syndrome dry eye (27). Which form of lacritin contributes to tear film stability? Anti-C-term lacritin antibody detects lacritin monomer, polymer, and C-terminal proteoforms—but not N-terminal proteoforms. Anti-N-term lacritin antibody 1F5 detects N-terminal proteoforms, monomer, and polymer, but not C-terminal proteoforms (Fig. 1*A*; (26)). We subjected normal human tears to 1F5 immunodepletion (Fig. 1*C*; Fig. S1*A*). Film stability under compression was unaffected per “lift off” area (∼48%; Fig. 1*D*(i), *D inset*, *right*) and maximum surface pressure (∼26 mN/m; Fig. 1*D*(ii), *D inset*, *left*) suggesting that C-terminal proteoforms maybe contributory. To test this possibility, we synthesized C-terminal synthetic peptides “N-94,” “N-94/C-6,” and “N-64/C-31” that together span all detected C-terminal proteoforms in tears (Fig. 1*A*) and therefore serve as surrogates. N-terminal 24 amino acid peptide “C-95” lacking 95 C-terminal amino acids (Fig. 1*A*) was included as a negative control. “N-94” represents lacritin's C-terminal 25 amino acids with two amphipathic α-helices and a six amino acid C-terminal random coil domain, whereas “N-94/C-6” lacks the latter. “N-64/C-31” is a more internal amphipathic α-helix ((29); Fig. 1*A*). In total, 6 μM of each was added to anti-C-term lacritin-depleted tears (Fig. 1*F*). In total, 6 μM is the suspected concentration of C-terminal lacritin proteoforms in human basal tears, *versus* ∼18 to 27 μM for all anti-Pep Lac N-Term detectable lacritin (39). N-94 and N-94/C-6 (“lift off” area: 41 and 49%, [Fig. 1, *G*–*H*]; maximum surface pressure: 23 and 24 mN/m; respectively) largely restored film stability under compression (Fig. 1, *G*–*H*) and expansion (Fig. S1*B*). Not beneficial were N-64/C-31 and C-95 (“lift off” area: 23 and 21%; maximum surface pressure: 17 and 11 mN/m (Fig. 1, *G*–*H*).

Lacritin monomer is selectively deficient in tears of almost all forms of dry eye (reviewed by Willcox *et al.* (40)). Are C-terminal proteoforms also lacking, and if so is their absence contributory to tear film instability? Dry eye is generally most severe in Sjögren's syndrome, an autoimmune form of dry eye disease (41). We tested basal tears from 21 normal or 21 non-Sjögren's dry eye individuals (Fig. 1*J* (i)) and tears collected without anesthesia from 21 Secondary Sjögren's syndrome dry eye patients (Fig. 1*J* (ii)). C-terminal proteoforms and monomer were deficient or absent in most non-Sjögren's and Sjögren's syndrome dry eye tears (Fig. 1*J*). Sjögren's syndrome samples were kindly provided by the Sjögren's International Collaborative Alliance for which volume was limiting. Non-Sjögren's dry eye tears (from the Warfighter Refractive Eye Surgery Program and Research Center, Fort Belvoir VA) were sufficient for compression and expansion isocycling. Compression values (“lift-off” area 26% [Fig. 1, *H*–*I*]; highest surface pressure 15%; Fig. 1*I*) were essentially identical to anti-C-term lacritin-depleted normal tears, as was the benefit of supplementation with 6 μM N-94 or N-94/C-6 (42–43% [Fig. 1, *H*–*I*] and 21–22 mN/m; Fig. 1*I*). Similar benefit was apparent in expansion profiles (Fig. S1*B*). Thus, lacritin C-terminal proteoforms, but not apparently lacritin monomeric or polymeric forms, lower tear surface tension and when selectively absent or deficient in dry eye tears contribute to decreased film stability upon compression that can be largely rescued with N-94 or N-94/C-6.

### C-terminal lacritin peptide rescue restores elasticity

**Figure 2:**
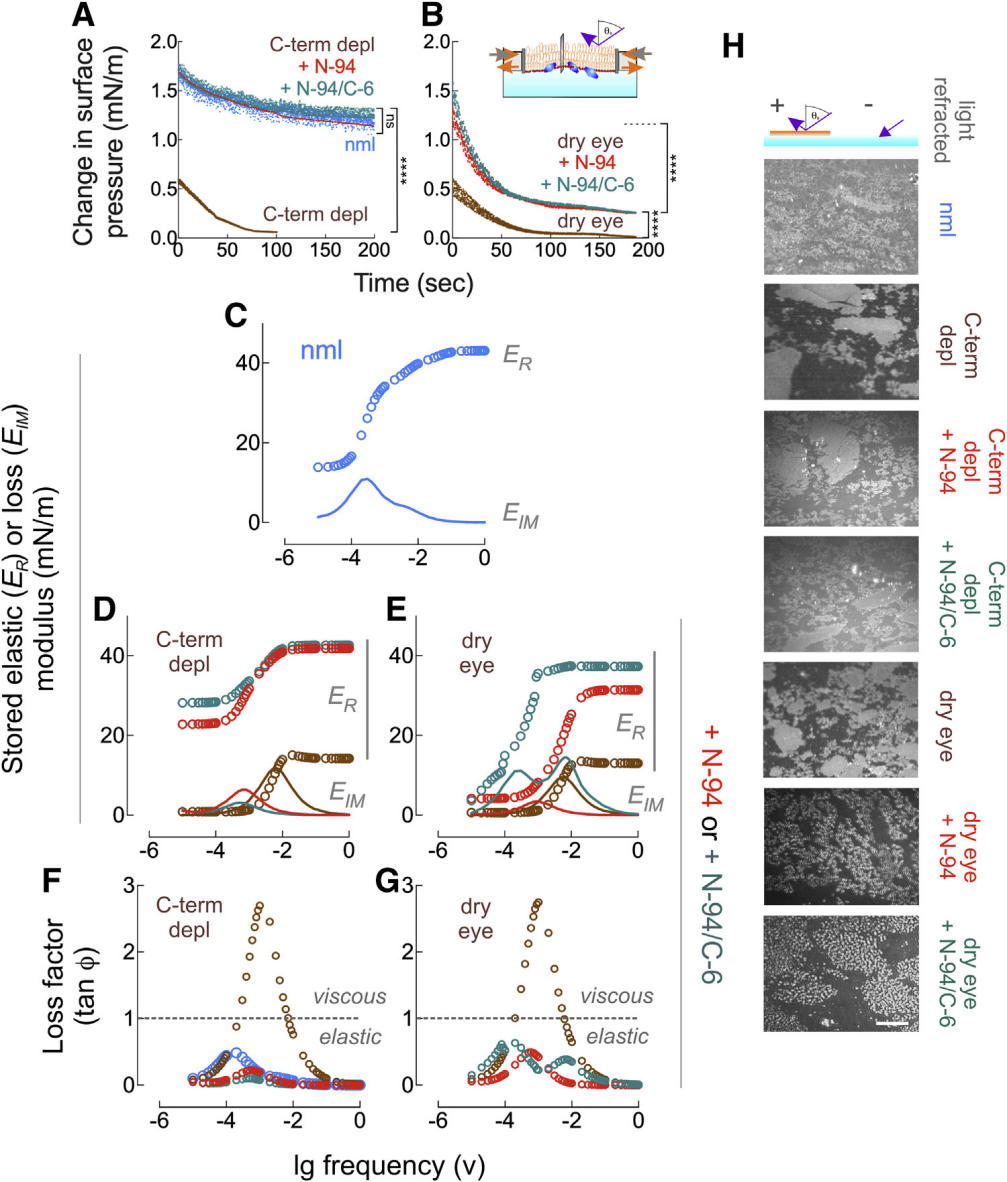
**Tear film stabilizing activity of lacritin C-terminal proxy proteoforms N-94 and N-94/C-6 restore normal viscoelasticity.***A*, time-dependent relaxation of surface tension after pre-equilibrated anti-C-term lacritin-depleted (without or with 6 μM N-94 or N-94/C-6) or normal human basal tear films formed as per Figure 1 were subjected to a sudden step compression of less than 5% of the prior surface area. ∗∗∗∗*p* < 0.0001; ns, not significant [two-way ANOVA with Sidak multiple comparisons test]. *B*, same procedure applied to human dry eye basal tear films (without or with 6 μM N-94 or N-94/C-6). For both *A* and *B*, shown are individual replicates for each experiment performed in triplicate (>2300 data points per variable with the exception of anti-C-term lacritin-depleted tears plus 6 μM N-94 [one replicate] and anti-C-term lacritin-depleted tears alone [1260 data points]). ∗∗∗∗*p* < 0.0001 with top bracket a comparison to normal [two-way ANOVA with Sidak multiple comparisons test]. *Inset*, schematic diagram of time-dependent relaxation. *C*–*G*, Fourier transform (44) of the relaxation data in *A*–*B* as the stored elastic (*E*_*R*_) or loss (*E*_*IM*_) moduli, or loss factor (tan ϕ)—both as a function of the log frequency. Color code of data is per *A*–*B*. *C*, stored elastic and loss moduli of normal human basal tears. *D*, stored elastic and loss moduli of anti-C-term lacritin-depleted human basal tears without or with N-94 or N-94/C-6 supplementation. *E*, stored elastic and loss moduli of human dry eye basal tears without or with N-94 or N-94/C-6 supplementation. *F*, loss factor of normal human basal tears *versus* anti-C-term lacritin-depleted tears without or with added 6 μM N-94 or N-94/C-6; horizontal line: values less than or greater than 1 are respectively elastic or viscous. *G*, loss factor of human dry eye basal tears without or with added 6 μM N-94 or N-94/C-6. *H*, light incident to the Brewster angle reflects in a manner corelative to regional lipid thickness (top schematic). Shown is the reflection off normal, anti-C-term lacritin-depleted (without or with 6 μM N-94 or N-94/C-6), or dry eye (without or with 6 μM N-94 or N-94/C-6) basal tear films. Bar = 1 μm. Representative of triplicate experiments. Data for *A*–*G* in Data files Figure S2.

Viscoelasticity underlies tear compression and expansion properties that in turn modulate the quality of light refraction necessary for visual acuity—a feature compromised in dry eye (42). We monitored the time-dependent relaxation of surface tension after pre-equilibrated films at a surface pressure of ∼15 mN/m were subjected to a sudden step compression of less than 5% of the prior surface area (Fig. 2, *A*–*B*, inset). The initial maximal surface pressure as the instantaneous stress response differed substantially between normal (∼1.7 mN/m) and both anti-C-term lacritin-depleted normal (∼0.6 mN/m) and dry eye tears (∼0.6 mN/m; [Fig. 2, *A*–*B*]). As per compression/expansion isocycling, spiking N-94 or N-94/C-6 into anti-C-term lacritin-depleted normal tears was fully corrective (Fig. 2*A*), but only partially so in dry eye tears in which the initial reading was elevated (respectively ∼1.2 and 1.6 mN/m) but then fell off precipitously with relaxation (Fig. 2*B*). Viscoelasticity was assessed by Fourier transform of the relaxation data (43,44) thereby yielding the stored elastic modulus (*'E*_*R*_*'*) and loss modulus (*'E*_*IM*_*'*) (Fig. 2, *C*–*E*)—the latter a measure of viscous behavior. N-94 and N-94/C-6 elevated the stored elastic modulus and diminished the loss modulus of both anti-C-term lacritin-depleted normal tears and dry eye tears (Fig. 2, *D*–*E*). The “dilatational elastic modulus” (*“E∗”*) is the sum of *E*_*R*_ and *E*_*IM*_, and the quotient of *E*_*IM*_*/E*_*R*_, (“tan ϕ”) is the “loss factor” such that values less than or greater than 1 are respectively elastic or viscous. At all frequencies, normal tears (maximal loss factor 0.5) as well as N-94 or N-94/C-6 supplemented anti-C-term lacritin-depleted normal (respective maximal loss factors 0.2, 0.1) and supplemented dry eye tears (respective maximal loss factors 0.5, 0.6) are elastic (Fig. 2, *F*–*G*). This contrasts with anti-C-term lacritin-depleted normal and dry eye (respective maximal loss factor 2.7, 2.8) tears that are respectively viscous between 10^−3.5^ and 10^−2.2^ Hz, and 10^−3.7^ and 10^−2.3^ Hz (Fig. 2, *F*–*G*). Curious about the role of N-94/C-6 amino acids with nonpolar side chains, we synthesized “N-94/C-6-ser” in which seven of eight (not alanine) were replaced with serine. Serine is identical to alanine, but with polar –OH group. Also, serine is uncharged and neither hydrophobic nor hydrophilic. Lacritin-depleted tears supplemented with N-94/C-6-ser (maximal loss factor 0.3) were surprisingly elastic (Fig. S2), suggesting that prevention of rupture is not a property of hydrophobicity, but instead likely due to charge. Indeed, N-94/C-6 contains six amino acids with charged side chains at neutral pH (four lysines and two glutamic acids). To ask whether normalized viscoelasticity was manifested in tear film structure, we applied Brewster angle microscopy. Light incident to the Brewster angle reflects in a manner proportional to the square of the regional lipid thickness (45) such that white areas are thick and gray areas less so. Black areas lacking lipid islands are nonreflective (Fig. 2*H*, top schematic; Figs. S2 and S3), as per regions in anti-C-terminal lacritin-depleted and dry eye tears. Supplementation of anti-C-terminal lacritin-depleted tears with N-94 or N-94/C-6 yields images similar to normal tears, whereas the pattern is more complex after supplementation of dry eye tears with islands packed with globular structures. By quantitation, normal tears present as a single population, in contrast to three populations for anti-C-terminal lacritin-depleted tears and dry eye tears (Fig. S3). With N-94 or N-94/C-6 supplementation, these coalesce into two (anti-C-terminal lacritin-depleted) or one (dry eye) peaks. Two peaks were also observed in N-94/C-6-ser supplemented anti-C-terminal lacritin-depleted tears (Fig. S2). Thus N-94 and N-94/C-6 largely correct for loss of elasticity of dry eye tears by possibly acting as a surfactant, although the supplemented dry eye films are morphologically distinctive.

### C-terminal lacritin peptides interact with and stabilize meibomian gland secretions

**Figure 3:**
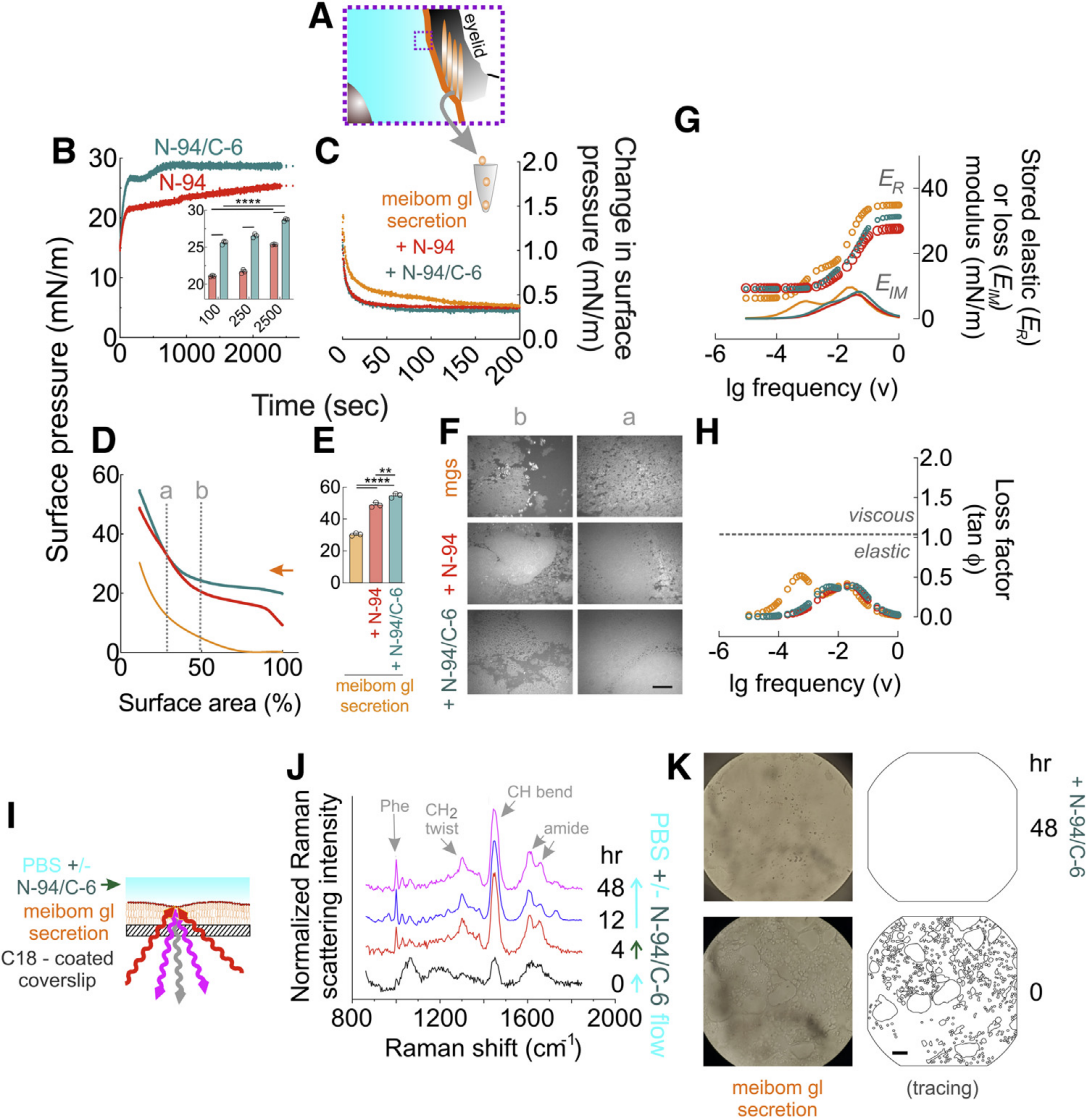
**Lacritin C-terminal proxy proteoforms N-94 and N-94/C-6 restore viscoelasticity in whole or part through rapid insertion into the tear lipid layer.***A*, schematic diagram of human basal tears from dashed boxed in Figure 1*C* with “meibum” lipid layer (*orange*) and underlying aqueous layer (*aqua*), and covering eyelid containing multiple meibomian glands from which meibum derives. *Small dashed box* indicates the region highlighted in Figure 4*B* (*inset*). Human meibum was collected by lid compression and dissolved in chloroform (1 mg/ml). *B*, 47 μg of human meibum spotted on a 80 ml PBS subphase was equilibrated for 15 min under cover while chloroform evaporated and then subjected to compression isocycling reaching 15 mN/m surface pressure at which time surface pressure was monitored as N-94 or N-94/C-6 was introduced into the subphase at a final concentration of 6 μM. Shown are individual replicates for each experiment performed in triplicate (>29,000 data points per variable). *Inset*, comparative surface pressure attained (mean with S.D [n = 3], ∗∗∗∗*p* < 0.0001; [two-way ANOVA with Sidak multiple comparisons test]). *C*, time-dependent relaxation of surface tension after pre-equilibrated human meibum films (without or with 6 μM N-94 or N-94/C-6) were subjected to a sudden step compression of less than 5% of the prior surface area. Shown are individual replicates for each experiment performed in triplicate (>8000 data points per variable). *D*, compression isotherm of meibum films (without or with 6 μM N-94 or N-94/C-6). Shown are individual replicates for each experiment performed in triplicate (2163–2166 data points per variable). *E*, comparative highest surface pressure attained (mean with S.D [n = 3], ∗∗∗∗*p* < 0.0001; ∗∗*p* = 0.0027 [one-way ANOVA with Tukey multiple comparisons test]). *F*, Brewster angle microscopy of human meibum films (without or with 6 μM N-94 or N-94/C-6) at low (*b*) or higher (*a*) pressure—as indicated in *D*. Bar = 1 μm. Representative of triplicate experiments. *G*–*H*, Fourier transform (44) of the triplicate relaxation data in *C*. *G*, stored elastic (*E*_*R*_) and loss (*E*_*IM*_) moduli of human meibum films without or with N-94 or N-94/C-6 supplementation. *H*, loss factor (tan ϕ) of human meibum films without or with added 6 μM N-94 or N-94/C-6; horizontal line: values less than or greater than 1 are respectively elastic or viscous. *I*, schematic diagram of Raman scatter of incident light (647.1 nm (82)) by 10 to 20 μm thick meibum film that had been dried on C18 coated coverslips and then subjected to PBS flow (0.2 ml/min) in the absence or presence of 6 μM N-94/C-6 (objective aperature of confocal Raman microscope not shown). *J*, Raman spectra collected as a function of time before (0 h), during (4 h), and after (12–48 h) N-94/C-6 accumulation, the latter detected by increases in the phenylalanine ring-breathing mode (1000 cm^−1^) and amide modes (1600–1700 cm^−1^ [n = 2; each performed in triplicate]). *K*, light microscopic images with adjacent tracing of the meibum film prior to (0 h) and 48 h after N-94/C-6 accumulation. Bar = 20 μm. Data for *B*–*E*, *G–H* in Data files Figure S3.

If N-94 and N-94/C-6 are capable of acting as a tear lipid surfactant, one likely destination is the aqueous/lipid interface thereby accommodating respective hydrophilic and hydrophobic faces of their two amphipathic α-helices—as per pulmonary surfactant protein B (32, 33, 34), although per N-94/C-6-ser the hydrophobic face appears to contribute little to stability. Tear lipids largely derive from eyelid meibomian glands, a form of sebaceous gland with characteristic holocrine secretion. To address this possibility directly, we collected and then pooled meibomian gland lipid secretions into chloroform (1 mg/ml; Fig. 3*A*) from four normal individuals for adsorption (Fig. 3*B*), relaxation (Fig. 3*C*), compression (Fig. 3, *D*–*E*)/expansion isocycling, Brewster microscopy (Fig. 3*F*; Fig. S4), and viscoelasticity (Fig. 3, *G*–*H*) studies—again all at 35 °C. We also performed Raman microscopy (Fig. 3, *I*–*K*) using in part meibum collected from 27 other normal individuals. N-94 and N-94/C-6 introduced into the PBS subphase rapidly penetrated into the overlying meibum film with kinetics (Fig. 3*B*; line fit R^2^ ≥ 0.96) in keeping with a two-step reaction model:

$$\text{A }\overset{\text{k}1}{\to }\text{ B }\overset{\text{k}2}{\to }\text{ C}$$

**Figure 4:**
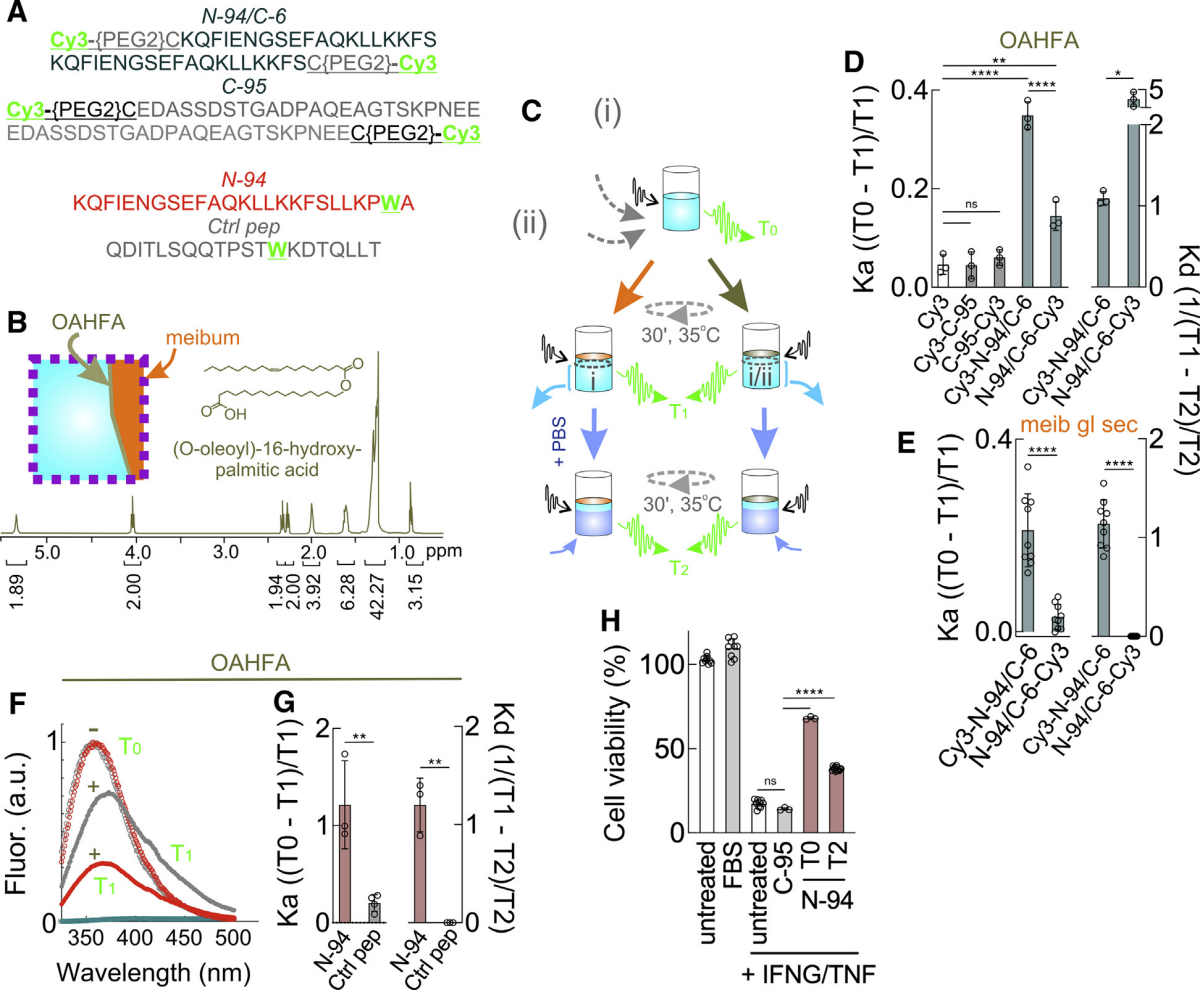
**Slow release of N-94 and Cy3-N-94/C-6 from floating, gently rotating OAHFA and meibum films.***A*, synthetic peptides with fluorescent Cy3 tag (i), or tryptophan (ii). *B*, ^1^H NMR spectrum (600 MHz; deuterated chloroform) of 16-(oleoyloxy)hexadecanoic acid (OAHFA) synthesized using the method of Balas *et al.* (49). *Inset left*, suspected location of OAHFA's at the aqueous (*aqua*)/lipid (*orange*) interface; for orientation, refer to box in Figure 3*A*. *Inset right*, linear structure of synthesized 16-(oleoyloxy)hexadecanoic acid. *C*, procedure with PBS and (i) or (ii) individual peptide-containing quartz cuvettes (actually rectangular) that were overlayed with OAHFA or meibum films before and after fluorescent excitation with intermittent rotation for 30 min. Two-thirds of the PBS were then replaced with fresh PBS, followed by rotation and fluorescent excitation. *D*, calculated Ka or Kd of Cy3 tagged peptides in cuvettes with OAHFA (left graph: mean with S.D [n = 3], ∗∗∗∗*p* < 0.0001; ∗∗*p* = 0.0021 [one-way ANOVA with Dunnett's multiple comparisons test]; *right graph*: mean with S.D [n = 3], ∗*p* = 0.0117 [unpaired t test]), or *E*, or with meibomian gland secretion (mean with S.D [n = 3], ∗∗∗∗*p* < 0.0001 [unpaired t test]). *F*, tryptophan emission spectra of N-94 or “Ctrl pep” in the absence or presence of OAHFA. *G*, calculated K_a_ or K_d_ of N-94 or Ctrl pep with OAHFA (mean with S.D [n = 3], ∗∗*p* = 0.0064 and 0.0016 [respectively *left and right graphs*; unpaired *t* test]). *H*, human corneal epithelial (HCE-T) cells without or stressed with interferon-gamma and tumor necrosis factor in the presence of 1.38 μM C-95, T0 N-94, or dissociated T2 N-94. 1.38 μM is the concentration of T2 N-94 released from OAHFA over 30 min (mean with S.D [n = 3], ∗∗∗∗*p* < 0.0001; ns, not significant [one-way ANOVA with Tukey's multiple comparisons test]). Data for *D*−*H* in Data files Figure S4.

in which *A* to *B* could be due to docking and *B* to *C* to incorporation. The equation describing such a mechanism (Fig. S5) derives respective N-94 and N-94/C-6 *k1* values of 2.678e^−02^ and 2.139e^−02^ s^−1^, as well as 3.434e^−04^ and 2.411e^−03^ s^−1^ for *k2*. The implication is that putative docking is more rapid than incorporation and that N-94/C-6 does the latter much more quickly than N-94 (Fig. 3*B*)—in keeping with N-94/C-6's superior performance in tear viscoelasticity studies (Fig. 2, *D*–*E*). Yet both peptides are film stabilizing, as per elevated compression values (Fig. 3, *D*–*E*), superior film thickness (Fig. 3*F*; Fig. S4), and a slight reduction in the maximal loss factor to 0.4 (lg frequency 1.5) from 0.5 (lg frequency 3.3; Fig. 3*H*). To confirm this interaction, we flowed 250 μM N-94/C-6 in PBS onto C_18_-functionalized fused-silica coverslips dosed with sufficient meibum to form a 10 to 20 μm film for analysis by Raman microscopy at 35 °C. Higher concentration N-94C-6 was necessary for detection. Raman microscopy monitors inelastic light scattering at frequencies less than or greater than incident light with the difference from incident known as a Raman shift (Fig. 3*I*). A Raman shift not prominent in meibum but characteristic of aromatic groups contributed by N-94/C-6's three phenylalanines provided evidence for incorporation (Fig. 3*J*) that in turn transformed meibum into a continuous, thicker film (Fig. 3*K*)—the latter in agreement with an increase in the meibum CH2-twisting mode at 1300 cm^−1^ (Fig. 3*J*). Thus, N-94 and N-94C-6, as surrogates for natural C-terminal lacritin proteoforms in normal tears but substantially lacking in dry eye tears, rapidly interact with and stabilize the tear lipid layer.

**Figure 5:**
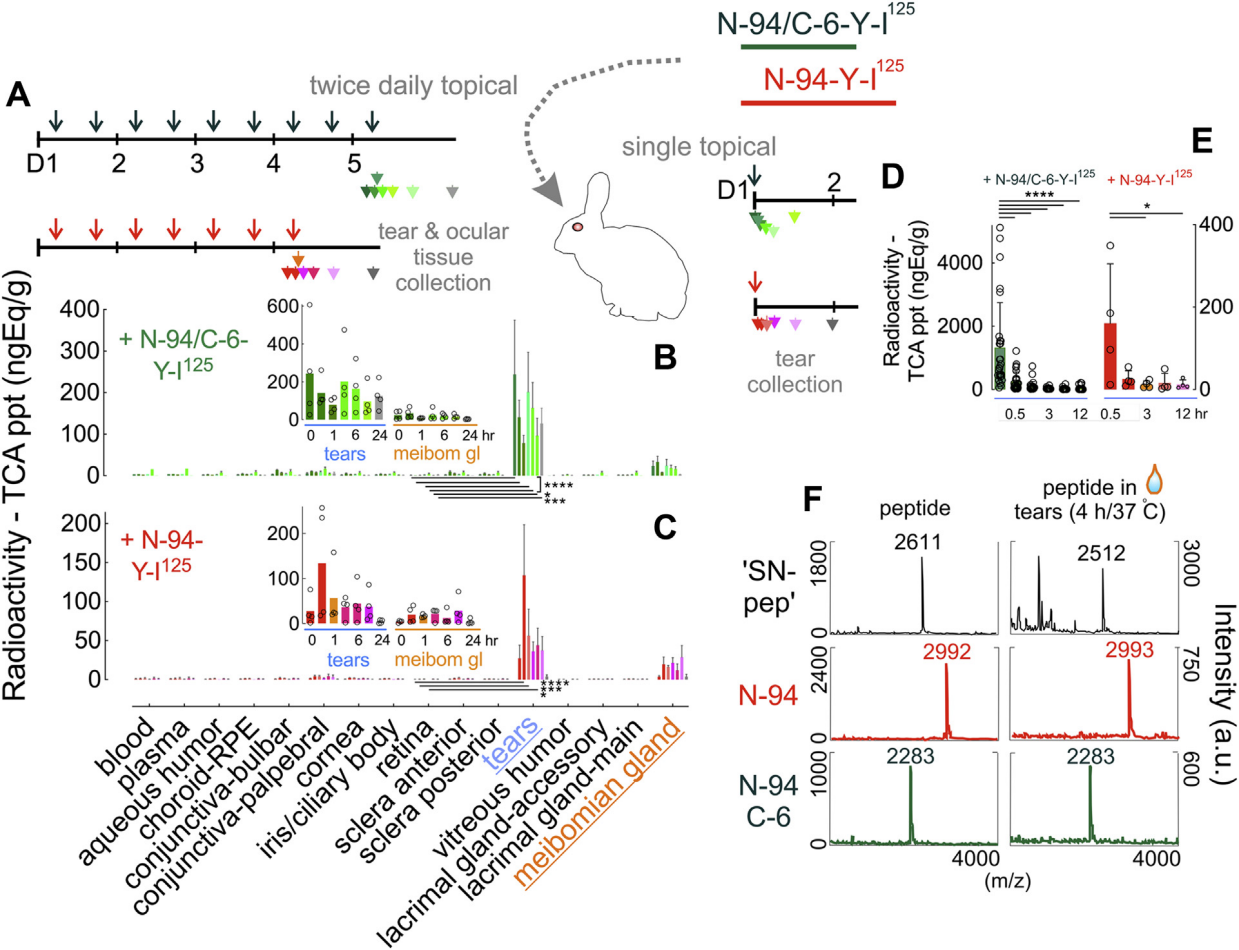
**Slow tear release of topically applied**^**125**^**I-N-94 and**^**125**^**I-N-94/C-6 from rabbit eyes.***A*, 35 μl (10 μCi) of 44 μM ^125^I-N-94/C-6 or 4 μM ^125^I-N-94 were topically added to each eye of respectively 14 rabbits twice daily for 4 (^125^I-N-94/C-6) or 3 (^125^I-N-94) days with the final treament on the morning of the fifth (^125^I-N-94/C-6) or fourth (^125^I-N-94) day (respective *dark green* or *red downward arrows*) after which tears were collected at 0.5, 1, 3, 6, 12, and 24 h postdose from two animals per time point who were then euthanized for collection of blood and ocular tissues (respective *green or red downward arrowheads*). *B*–*C*, TCA precipitable radioactivity of samples was assessed by scintillation counting and expressed per tissue wet weight. ∗∗∗∗*p* < 0.0001; ∗∗∗, respectively 0.0007 and 0.0003, ∗, respectively 0.0365 and 0.0198 (two-way ANOVA with Tukey's multiple comparisons test comparing tears *versus* retina); study was performed once each for rabbits treated with ^125^I-N-94 or with ^125^I-N-94/C-6). *D*, a single 35 μl (10 μCi) dose of 44 μM ^125^I-N-94/C-6 or 4 μM ^125^I-N-94 (as three 1.3 μM doses over 10 min) was topically added to each eye of respectively 14 or 12 rabbits. Tears were collected 0.25, 0.5, 1, 3, 6, or 12 h after. *E*, TCA precipitable radioactivity of samples from *D* were assessed as in *B*–*C* (∗∗∗∗*p* < 0.0001; ∗*p* = 0.0431 [one-way ANOVA with Tukey's multiple comparisons test]; study was performed once each for rabbits treated with ^125^I-N-94 or with ^125^I-N-94/C-6). *F*, 50 μM of N-94, N-94/C-6, or positive control “SN-pep” (56) was incubated for 4 h at 37 °C with 10 μl of human basal tears. Samples before (*left column*) or after tear treatment were assessed by MALDI mass spectrometry ([n = 1). Data for *B*–*E* in Data files Figure S5.

Surfactant protein B is thought to superficially associate with underlying anionic phospholipids of pulmonary surfactant (46), whereas surfactant protein C tilts into the membrane (47)—both to ease spreading during respiration. How might N-94/C-6- and N-94-like proteoforms associate and with what affinity in films not subject to prior restructuring by isocycling? We synthesized N-94/C-6, or negative control C-95, with polyethylene glycol-linked Cy3 thiol-coupled to an added N- or C-terminal cysteine (Fig. 4*A*). We then reconstituted peptides to 6 μM in PBS and overlaid each with meibum or (O-acyl)-ω-hydroxy fatty acid (OAHFA) film. OAHFAs are thought to reside at the meibum/aqueous interface (Fig. 4*B*, *inset*), in keeping with their amphiphilic (both lipophilic and hydrophilic) nature. Here they are presumed to contribute to an elastic monolayer (48) essential for tear stability. OAHFAs appear to be the only lipid class downregulated in dry eye (15), and transgenic mice lacking fatty acid ω-hydroxylase in the cornea and meibomian gland (and thus ω-O-C16:1 OAHFA's and type 2ω wax diesters) develop a form of dry eye characterized by increased blinking, corneal damage, meibomian orifice plugging, and decreased tear breakup time although tearing is normal (17). We synthesized the OAHFA 16-(O-oleoyloxy)hexadecanoic acid (also known as 16-(O-oleoyloxy)palmitic acid; Fig. 4*B*) as per Balas *et al.* (49) and estimated that ∼14.3 × 10^13^ 16-(O-oleoyloxy)hexadecanoic acid molecules should be required to cover a PBS subphase with a surface area of ∼9.5 × 10^13^ nm^2^ assuming ∼1.5 molecules per nm^2^—as determined for dipalmitoylphosphatidylcholine or phosphatidylcholine (50). Prior to overlaying OAHFA or meibum onto the PBS subphase, we measured the fluorescence of Cy3-N- or -C-terminal labeled N-94/C-6 or C-95 in PBS as the “T0” values (Fig. 4*C*). Topping this with OAHFA or meibum under gentle rotation for 30 min at 35 °C tested the affinity of peptides for each film, as inversely reflected by the level of Cy3 fluorescence in the PBS subphase (“T1”) according the equation below. K_a_ is the association constant:

$${\text{K}}_{\text{a}}\;=\;\frac{\text{T0}-\text{T}1}{\text{T1}}$$

K_a_'s of Cy3-N-94/C-6 (respectively 0.35 ± 0.03 and 0.21 ± 0.04 for OAHFA and meibum) exceeded those of N-94/C-6-Cy3 (0.14 ± 0.03 and 0.03 ± 0.03) by 2.5- to 7-fold (Fig. 4, *D*–*E*), implying preferential association of the C-terminal half. This is in keeping its C-terminal net positive charge (pI of 10.3 *versus* 4.6 for C-terminal ten amino acids *versus* nine N-terminal amino acids) and superior hydrophobicity (five *versus* three amino acids with nonpolar side chains). In contrast, C-95 Ka's were at background levels similar to Cy3 alone (Fig. 4*D*).

While not (or minimally) disturbing the OAHFA or meibum film, we next replaced 2/3 of the subphase with fresh PBS (Fig. 4*C*). Our purpose was to ask whether peptide dissociation from OAHFA or meibum was detectable although not apparent by Raman microscopy (Fig. 3*J*) nor by Langmuir surface balance (not shown). After 30 min of gentle rotation at 35 °C, Cy3 fluorescence of the PBS subphase was assessed (“T2”). The dissociation constant (K_d_) of N-94/C-6 was estimated as:

$${\text{K}}_{\text{d}}\;=\;\frac{1}{\left(\text{T}1\;-\text{ T}2\right)\text{/T}2}$$

This suggested a K_d_ of 1.1 from meibum and OAHFA (Fig. 4, *D*–*E*).

For validation, advantage was taken of N-94’s penultimate C-terminal tryptophan (Fig. 4*A*) that is accordingly absent from N-94/C-6. In PBS, N-94 displays a fluorescence optimum of 361 nm after excitation at 280 nm implying exposure (51) appropriate for quenching. Tryptophan quenching in meibum was impractical with background expected from its numerous apparent protein constituents—including lacritin (52). Chloroform is a polar solvent often employed in partition studies with aqueous solutes. When 6 μM N-94 was added for 30 min to PBS overlying a chloroform subphase at 34 °C, ∼72 ± 10% became quenched (Fig. S6*A*) and a thin film formed at the interface suggesting affinity but inability to partition. Studies were accordingly performed with OAHFA as described above in which 52% of N-94 and only 15% of equimolar “Ctrl pep” were quenched (Fig. 4*F*). This corresponded to respective (T0 − T1)/T1 K_a_'s of 1.21 ± 0.45 and 0.2 ± 0.09 and an N-94 1/(T1 − T2)/T2 K_d_ of 1.20 ± 0.27 (Fig. 4*G*). “Ctrl pep” was syndecan 1 “Pep30-50” peptide (53), also with a single tryptophan (Fig. 4*A*) but with PSIPRED-predicted random coil structure and fewer amino acids with nonpolar (30 *versus* 44%) or basic (5 *versus* 25%) side chains. The fluorescent contribution of 1/(T1 − T2)/T2 disassociated N-94 was calculated *via* the equation below that takes into consideration peptide in residual PBS. Dissociated N-94 was intact by matrix-associated laser desorption/ionization (MALDI) mass spectrometry (Fig. S6*B*) and restored homeostasis to interferon-γ and TNF-stressed human corneal epithelial cell cultures (Fig. 4*H*), as performed after calculating the quantity released per the equation:

$$\left[{\text{K}}_{\text{a}}^{\left(\text{T0}-\text{T}1\right)}\times {\text{K}}_{\text{d}}^{\left(\text{T}1-\text{T}2\right)}\times {\text{fluorescence}}^{\text{T}1}-\left(0.33\right){\text{fluorescence}}^{\text{T}1}\right]\text{/}\left[{\text{K}}_{\text{d}}^{\left(\text{T}1-\text{T}2\right)}+1\right]$$

Molar amounts were then obtained by coupling this value to the extinction coefficient, as confirmed by immunodot blot analysis (Fig. S6*C*). Thus N-94 and N-94/C-6, as surrogate C-terminal lacritin proteoforms, appear to preferentially associate with meibum and OAHFA of the tear lipid layer through their C-termini and (at least in nonstructured films) are subject to release at a level not detected by Raman microscopy (Fig. 3*J*) nor by Langmuir surface balance (not shown) but sufficient to restore epithelial homeostasis.

### Release time slows with repeated topical application

Topical application of 5-Dodecanoylaminofluorescein (94 mM) and sodium fluorescein (0.13 mM) onto human eyes suggests respective turnover rates of 0.93 ± 0.36% and 10.3 ± 3.7% per minute, respectively (54) over a total of 108 ± 39 and 9.7 ± 4 min from respective lipid and aqueous portions of tears. The former value can also be considered the lipid release time. We synthesized N-94 and N-94/C-6 each with an added C-terminal tyrosine for iodination and performed ocular and systemic pharmacokinetic studies in rabbits. Human meibum and rabbit meibum display significant compositional differences, likely in keeping a much slower blink rate and greater tear stability in rabbits. Nonetheless, both contain OAHFA (55). By assessing residence time in tears, information can be gained on tear lipid affinity *in vivo*. 4 μM ^125^I-N-94 or 44 μM ^125^I-N-94/C-6 were applied twice daily to rabbit eyes for 3 and 4 days, respectively (Fig. 5, *A*–*C*), or respectively as three 1.3 μM doses over 10 min or as a single 44 μM dose (Fig. 5*D*). Concentrations chosen were in support of a phase 2 human trial. Turnover was exponential with data best fitting the equation:

$$\text{R }=\;{\text{R}}_{\text{0}}\;+\text{ A.EXP}\left[-\text{t/}\tau \right]$$

where R = radioactivity, R_0_ = baseline radioactivity, A is a coefficient, t = time and τ = release (turnover) time. Accordingly, N-94 and N-94/C-6 release times were respectively 400 ± 95 and 2000 ± 500 min for multiday dosing, and 13 ± 2 and 22 ± 4 min following 10 min of dosing. The same was true for the meibomian gland although at lower levels that were not statistically different from baseline (Fig. 5, *B*–*C*). Radioactivity in other tissues and fluids remained near or at baseline (Fig. 5, *B*–*C*). Thus, tear release (presumably from lipid) of ^125^I-N-94 and ^25^I-N-94/C-6 is respectively 4- to 20-fold slower than 5-Dodecanoylaminofluorescein, although only after repeated application. Repeated application achieved respective ^125^I-N-94 and ^25^I-N-94/C-6 totals of 1.1 or 14 nmol (seven or nine 35 μl doses of 4 or 44 μM). This contrasts with a single 94 nM dose of 5-dodecanoylaminofluorscein (one 1 μl dose of 94 mM (54)). If N-94 and N-94/C-6, as proxy for lacritin proteoforms, have a long residence time in tears, are they resistant to tear proteases? We incubated 50 μM N-94, N-94/C-6, or as positive control similar-sized laminin “SN-peptide” ((56); predicted β-sheet) with normal basal tears for 4 h at 37 °C. By MALDI mass spectrometry, only N-94 and N-94/C-6 remained intact (Fig. 5*F*)—perhaps aided by their helical structure that makes backbone amide bonds less accessible to proteases and/or by relevant inhibitors resident in tears. Thus N-94 and N-94C-6 are relatively protease-resistant, a property possibly enhanced by their association with the tear lipid layer.

### Tear serine proteases, aminopeptidases, and metalloproteinases may contribute to the generation of C-terminal lacritin proteoforms

**Figure 6:**
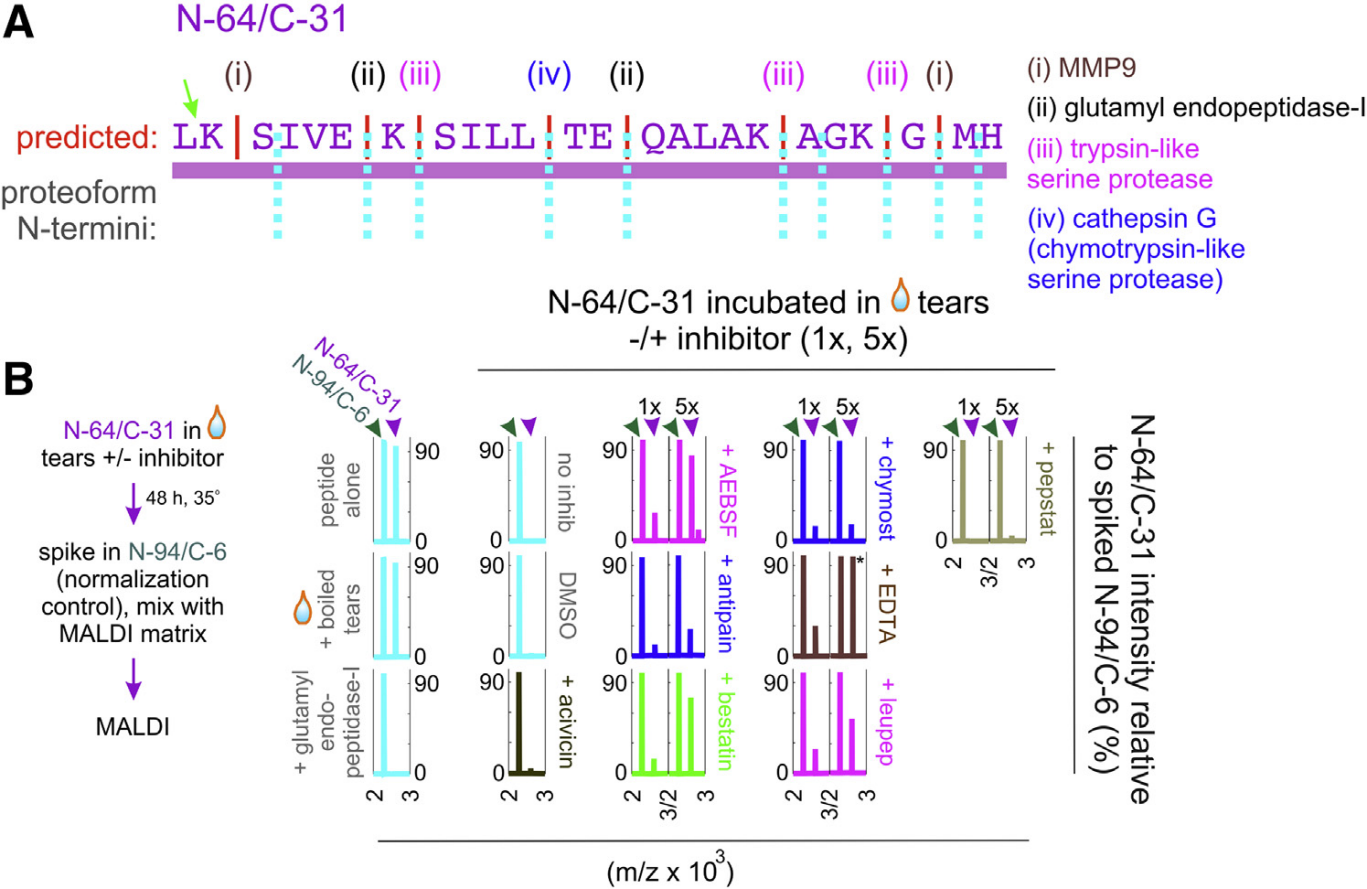
**Tear serine proteases, aminopeptidases, and metalloproteinases may contribute to the generation of C-terminal lacritin proteoforms.***A*, N-64/C-31 with predicted protease cleavage sites as N-termini for proteoforms. *B*, *left*, N-64/C-31 digestion and MALDI mass spectrometry analysis scheme using 38 μl of pooled basal tears. *Right*, N-64/C-31 MALDI profiles normalized to N-94/C-6 spiked in postdigestion. Incubations included two different concentrations of AEBSF (0.2 or 1 mM), antipain (74 or 370 μM), bestatin (130 or 650 μM), chymostatin (100 or 500 μM), EDTA (2.5 or 5 mM), leupeptin (10 or 50 μM), and pepstatin (1 or 5 μM). Representative of three optimization experiments.

C-terminal processing of lacritin's putative homolog dermcidin is the responsibility of extracellular carboxypeptidases, an endopeptidase and the aspartyl protease cathepsin D—all in human sweat (57). The outcome is the bactericidal peptide “SSL-25.” Tear proteases include: alanyl aminopeptidase, arginyl aminopeptidase, complement factor B, cathepsins (B, D, G, S), dipeptidyl-peptidase 4, HtrA serine peptidase 1, matrix metallopeptidase-9 and -10, plasma kallikrein, plasminogen (plasmin), serine protease 8, serine carboxypeptidase, coagulation factor II (thrombin) and trypsin 1 (39). Tears are also rich in protease inhibitors (39). Of lacritin's 42 known C-terminal proteoforms (30), 22 (including the longest) share N-termini residing within the lacritin sequence “LKSIVEKSILLTEQALAKAGKGMH” represented by synthetic peptide N-64/C-31 (Figs. 1*A* and 6*A*; amino acids 65–88 of lacritin's 119 amino acids). *In silico* analysis by PROSPER (58) predicts LK|SIVE and AGKG|MH cleavage by matrix metallopeptidase-9 (M10.004) and SILL|TEQA by chymotrypsin-like serine protease cathepsin G (S01.133). N-64/C-31 is also predicted (ExPASY PeptideCutter (59)) to be sensitive to the serine protease glutamyl endopeptidase I (S01.269; SIVE|KSIL and LLTE|QALA) from eye commensal *S. epidermidis* and pathogen *S. aureus*, and to trypsin-like serine proteases (IVEK|SILL; ALAK|AGKG; KAGK|GMH). To assess which ones may contribute to processing, we first optimized tear volume such that all N-64/C-31 (50 μM) was fully hydrolyzed by 48 h at 35 °C and neutral pH (Fig. 6*B*). The assay was then repeated in the absence or presence of eight different proteolytic inhibitors at standard (1×) or fivefold higher concentrations (except EDTA at 0.5 and 1x) for endpoint analysis by semiquantitative MALDI mass spectrometry in which 4 μM N-94/C-6 was spiked into the digest immediately before mixing with MALDI matrix sinapinic acid (60). Processing was inhibited in a dose-dependent manner by AEBSF, bestatin, EDTA, leupeptin, or fully by boiling. Not effective were acivicin, antipain, chymostatin, and pepstatin. This is in keeping with involvement of tear cysteine proteases of the C1 and C2 families, metalloproteinases of the M1 and M10 B families, and serine proteases of the S1 family. Candidate N-64/C-31 tear proteases therefore include: cathepsin B (C1); calpain (C2); alanyl aminopeptidase, arginyl aminopeptidase (M1); MMP9, MMP10 (M10 B); cathepsin G, plasma kallikrein, plasmin, thrombin, and trypsin (S1).

## Discussion

How and which proteins help prevent premature collapse of the complex lipid film at the air/liquid interface of the eye necessary for vision? Using proxy synthetic peptides N-94 and N-94/C-6, we report that proteoforms from the C-terminus of the tear glycoprotein lacritin are essential through their rapid and stable insertion into tear lipids, including C16:1 OAHFA presumed to reside at the lipid/aqueous interface. This minimizes the loss factor between 10^−3.7^ and 10^−2.3^ Hz as a measure of viscosity by restoring elasticity to dry eye tears that otherwise are subject to premature collapse. We further report that C-terminal lacritin proteoforms are selectively deficient in dry eye. Interestingly, as proxy proteoforms gradually cycle off OAHFA in nonpressured films, bioactivity sufficient to restore homeostasis of corneal epithelial cells is retained—a slow release role for extracellular lipid films never previously appreciated.

C-terminal lacritin proteoforms were first noted as proteolytic fragments by western blotting of human tears (26) and in tear bactericidal assays leading to the discovery of cleavage-potentiated C-terminal “N-104” (29). This was later validated by Azkargorta *et al.* (30) through top-down sequencing of tears and identification of smaller “N-106” and “N-107” proteoforms that were also bactericidal. Their discovery of at least 40 additional C-terminal lacritin proteoforms of increasing size (Fig. 1*B*) paralleled a smaller library of lacritin synthetic peptides and recombinant fragments previously generated to dissect lacritin's ocular mitogenic (61), prosecretory (21, 22, 23, 24, 62), prohomeostatic (25), and cleavage potentiated bactericidal (29, 30) activities. All focused attention on lacritin's two C-terminal amphipathic α-helices required for ligation of heparanase-modified cell surface syndecan-1 (53, 63) necessary for epithelial targeting. Their similarity to N- and C-termini of processed lipid stabilizing pulmonary surfactant protein B (32, 33, 34), together with lacritin's detection in broncheoalveolar lavage (19, 20), and selective lacritin and C-terminal proteoform deficiency in dry eye were rationale for testing with tear lipids.

Early studies of floating lipid films with an air interface debated whether nonmucinous glycoproteins (64), or any protein (65), contributed substantially to integrity whereby rupture is minimized at low and high shear rates in a characteristic non-Newtonian viscoelastic manner (66). Later attribution of lung alveolar collapse in newborns to a variety of genetic mutations (35) and biophysical analyses with both lung surfactant proteins and candidate tear proteins (albumin (67, 68), keratin (69), lipocalin-1 (51, 67, 68, 70, 71), lactoferrin (67, 68, 70), lysozyme (67, 68, 70, 72)) validated how non-Newtonian behavior necessary to resist rupture is a consequence of complex protein–lipid interactions. Much remains to be learned about such interactions. Lipocalin-1 (10 μM) alone with captured lipid or mixtures of lactoferrin (21 μM) and lysozyme (136 μM) are non-Newtonian (70), yet tears without lipids are not (70). Soluble bovine ocular mucins can interact with and stabilize tear lipids (73, 74), but alone lack non-Newtonian behavior at physiological concentrations (73). That tear lipid binding proteins may functionally interact in modules, as suggested *via* Cytoscape for lacritin with lipocalin-1, apolipoprotein, lactoferrin, and others (75) is intriguing, although direct binding of these is not apparent in BioGrid. A coupled immunodepletion/rescue approach offered the novel opportunity to explore complex films and fluids in an otherwise undisturbed condition, making possible discovery of and dissection of lacritin's contribution through C-terminal proteoforms to the stability of whole tears and to tear lipids.

That C-terminal lacritin proteoforms are tear stabilizing aligns with changes of the tear lipid spreading rate and stability in health and disease (76) as it is well known that tightly packed, water insoluble lipid films (and hence those with higher lift-off areas and maximum surface pressure) are in general more elastic and able to recover after blink-like deformation (77). Further, the capacity of C-terminal proteoforms to restore elasticity is in keeping with dilatational properties of surfactant layers that define the resistance of the air/water surface of wetting tear, alveolar (78), or other films to extensional deformations caused by capillary waves or hydrodynamic phenomena and play a key role in the overall stability (79). Indeed dilatational rheology differed substantially (44, 80) between meibum from healthy individuals and from patients with meibomian gland disease. This was also true for contact lens lipid extracts collected from Caucasians *versus* those from Asians—the latter with a higher risk for dry eye disease. Thus, C-terminal proteoforms act as surfactants to promote tear viscoelasticity. As surfactants, they also reduce surface tension toward maintenance of a stable, “healthy” tear film. Stability suffers from lacritin downregulation in dry eye and would be further exacerbated by loss of OAHFA at the lipid/aqueous interface.

## Experimental procedures

### Synthetic peptides

“N-94” (KQFIENGSEFAQKLLKKFSLLKPWA) and “N-94/C-6” (KQFIENGSEFAQKLLKKFS), respectively representing the C-terminal active 25 or 19 amino acids of human lacritin (Fig. 1*A*) were manufactured by PolyPeptide Group (San Diego, CA) with amino terminal acetylation and carboxy terminal amidation (N-94/C-6; *i.e.*, absent of 94 N-terminal and 6 C-terminal amino acids) or only amino terminal acetylation (N-94), both under GMP conditions, and with trifluoracetic acid removed in place of acetate. Purity was respectively 98.9 and 97.2%. Numbering of synthetic peptides (Fig. 1*A*) and recombinant proteins excludes the signal peptide. N-94/C-6-Y and N-94-Y with added C-terminal tyrosine, control lacritin peptides N-64/C-31 (LKSIVEKSILLTEQALAKAGKGMH) with amino terminal acetylation and carboxy terminal amidation and C-95 (EDASSDSTGADPAQEAGTSKPNEE) with carboxy terminal amidation, as well as additional N-94 and N-94/C-6—both with terminal modifications as noted above—were synthesized by Genscript (Piscataway, NJ) and completed as acetate salts with respective purity of 95.9, 95.4, 98.1, 97.9, 95.7, and 96.4%. Also, Genscript synthesized Cy3-labeled N-94/C-6 (Cy3-{PEG2}CKQFIENGSEFAQKLLKKFS [“Cy3-N-94/C-6”] with amidated C-terminus and KQFIENGSEFAQKLLKKFSC{PEG2}-Cy3 [“N-94/C-6-Cy3”] with acetylated N-terminus, as well as as Cy3-labeled C-95 (Cy3-{PEG2}CEDASSDSTGADPAQEAGTSKPNEE [“Cy3-C-95”] with amidated C-terminus and EDASSDSTGADPAQEAGTSKPNEEC{PEG2}-Cy3 [“C-95-Cy3C”] as trifluoracetic acid salts with respective purity of 95, 97.2, 95.1 and 97%. Syndecan-1 peptide “Pep30-50” (QDITLSQQTPSTWKDTQLLT; (53)) and laminin peptide “SN-peptide” (SINNNRWHSIYITRFGNMGS; (56)) - all with amino terminal acetylation and carboxy terminal amidation were synthesized as trifluoracetic acid salts with respective purity of 97.6, 96.4, and 96.1%. All synthetic peptides were validated by electrospray ionization mass spectrometry. Aliquotted synthetic peptides were stored lyophilized at −70 °C in a dry environment.

### Tears, antibodies, meibum, OAHFA synthesis, Langmuir surface balance experiments, and Raman microscopy

Collection of all human samples was approved by Institutional Review Boards as specified below and abides by the Declaration of Helsinki ethical principles. Eighty-five basal tear samples from over 50 individuals (median age 30.2 years; 54% female) were collected after 0.5% proparacaine anesthesia from both eyes by wicking onto a paper “Schirmer” strip that had been carefully inserted under the center lower lid of the eye for 5 min. Wicking of >15 mm or ≤6 mm of tears was respectively considered normal or evidence of dry eye. Tears on strips were immediately stored at −80 °C. Approval was from the Walter Reed National Military Medical Center Institutional Review Board with informed consent. Tears collected on Schirmer strips without anesthesia from 21 patients with Secondary Sjögren's Syndrome were kindly provided by the Sjögren's International Collaborative Clinical Alliance (University of California, San Francisco) with Institutional Review Board approval at each of the Alliance collection sites. Only samples that had wicked ≤6 mm were tested. Tears were eluted from Schirmer strips immediately prior to experiments, by soaking in 25 μl of ice-cold PBS with protease inhibitors (Roche Complete Mini Inhibitor Cocktail; Sigma Chemical Co, St Louis MO) for 30 min on ice, and subsequent centrifugation at 20,000*g* for 30 min. A pool of 50 collected tears was subdivided into four pools. One was passed over a preimmune rabbit Ig column (“mock depletion”). Others were subjected to lacritin immunodepletion with rabbit polyclonal antibody “ab C-term” with specificity for lacritin's C-terminal 54 amino acids (26), as previously performed (25, 26). Another pool of five collected tears was subject to immunodepletion with mouse monoclonal antibody 1F5 directed against lacritin N-terminal synthetic peptide DPAQEAGTSKPNEEIS (amino acids 11–26). 1F5 (144 μg IgG1) or 432 μg ab C-term or preimmune rabbit Ig in 120 μl was immobilized on 0.2 ml protein A/G spin columns (Thermo Nab #89950, Thermo Scientific, Rockford, IL) using end-over-end mixing for 10 min at room temperature. Columns were washed with 20 bead volumes of 200 μl each of binding buffer and then similarly incubated end-over-end with pooled normal tears (315 μl/column) for 18 h at 4 °C. The “lacritin N-term-” and “lacritin C-term-depleted” or mock-depleted tear flow through were collected by centrifugation (5,000 x *g*, 1 min at 4 °C) with validation by ab C-term western blotting using LI-COR. No column leaching of antibody was detected by secondary alone western blotting. Dry eye, normal, or normal depleted tears were lyophilized for shipment on dry ice (−79 °C) for Langmuir surface balance experiments. Also shipped in this manner were lyophilized N-94, N-94/C-6, N-64/C-31, and C-95. Quantitation of lacritin monomer and C-terminal proteoforms was done by densitometery on tear Western blots immunostained with “anti-C-term” lacritin antibodies. All data were normalized to the maximum monomer per blot.

Meibum was collected from four normal individuals under the auspices of the Kyoto Prefectural University of Medicine Institutional Review Board (per a collaboration with GAG) and 27 other normals per the University of Virginia Insitutional Review Board, both with informed consent. For this purpose, the lower lid was squeezed using opposing cotton applicators or meibomian gland expressor forceps. Collection was into glass vials with 500 μl chloroform (1 mg/ml final concentration) for flash freezing on dry ice and storage at −80 °C.

16-(O-oleoyloxy)hexadecanoic acid (also known as 16-(O-oleoyloxy)palmitic acid) was synthesized as described by Balas *et al.* (49) using a two-step process involving esterification of oleic acid with 1,16-hexadecanediol followed by oxidation of the primary hydroxy group to a carboxylic acid. Purity by proton NMR was ≥95% with a yield of 1.41 gm. ^1^H NMR spectra were recorded on a Varian Inova 600 (600 MHz) spectrometer in CDCl_3_ with chemical shifts referenced to internal standards (CDCl_3_: 7.26 ppm ^1^H).

Langmuir surface balance experiments were performed as previously described (81) using a computer controlled Microtrough XL (Kibron, Helsinki Finland) with a 40 ml volume, an area of 225 cm, and a Wilhelmy wire probe with sensitivity exceeding 0.01 mN/m. Briefly, lyophilized intact normal or dry eye or N-term- or C-term-lacritin-depleted or mock-depleted pooled tears were reconstituted to their initial eluted volume in PBS with water. C-term-lacritin-depleted tears were also reconstituted with added N-94, N-94/C-6, N-64/C-31, or C-95 at a final peptide concentration of 6 μM. Three microliters of tears or 47 μg of pooled meibum, each without or with N-94 or N-94/C-6, were gently deposited as small submicro droplets at the air/PBS surface prewarmed to 35 °C, the corneal surface temperature (36). After equilibration for 15 min under cover to minimize evaporation and to keep dust-free, and with a continuous supply of ozone-depleted air, the film was subjected to experimental manipulation. After advancing or retreating dual opposing barriers at 1.37 cm^2^ per second over ten consecutive cycles, surface pressures were compared during compression and expansion isocycling. In other experiments, time-dependent relaxation of surface tension was monitored after pre-equilibrated films at a surface pressure of ∼15 mN/m were subjected to a sudden step compression of less than 5% of the prior surface areas. Fourier transformation made possible comparative appreciation of dilatational elastic moduli as per Loglio *et al.* (43). Further analyses were performed by Brewster angle microscopy (UltraBAM; Accurion GmbH, Göttingen Germany) to distinguish film regions by depth and continuity and *via* the pendant drop technique in which 2 μl of normal or lacritin-depleted tears were allowed to equilibrate for ∼3 min in a saturated vapor measurement cell to prevent evaporation, after which surface pressure was monitored for 60 s. Brewster angle microscopy images were analyzed by ImageJ.

For Raman confocal microscopy, 10 to 20 μm meibum films were created by application of 10 μl of 10 mg/ml meibum in chloroform to a coverslip on a 1 mm × 7 mm flow channel (82) followed by drying under nitrogen and then briefly under vacuum with thickness between coverslip–meibum and meibum–buffer interfaces estimated by incident laser beam reflection. The Raman microscopic probe (83, 84) has a depth resolution of ±1.2 μm (FWHM), a diameter of ∼500 nm, and a probe volume of ∼500 fl, which is well-matched to the meibum film deposited on the coverslip interface. For analyses, the probe was brought to 1.5 μm below the meibum–buffer interface with spectra collected at a laser power of 100 mW with an integration time of 2 min. After wetting for 10 min with PBS, a meibum spectrum was collected, followed by flow of 250 μM N-94/C-6 in PBS at 0.2 ml/min.

### *In vitro* and *in vivo* release kinetics

Cy3 fluorescence signals (excitation = 550 nm; detection = 570 nm) of 100 μl of Cy3 or Cy3-labeled N-94/C-6 (N- or C-terminal labeled) or Cy3-labeled C-95 (N- or C-terminal labeled) in PBS were collected in a black 96-well plate at 35 °C in a SpectraMax M3 microplate reader. Low photomultiplier tube power and 1 flash per read (reading from bottom) were applied to minimize bleaching. Wavelength cutoff was 570 nm. After a T0 fluorescence value of 6 μM peptide in PBS was obtained, 6 μM peptide in PBS was added to a final volume of 750 μl in a glass tube. The fluid surface area was 95 mm^2^, similar to the corneal surface area of ∼132 mm^2^. Onto this, 10 μl of 1 mg/ml OAHFA or meibum in acetone was allowed to spread, followed by rotation at 100 rpm for 30 min (35 °C). After removal of 500 μl of the PBS subphase for determination (in a 100 μl aliquot) of the T1 fluorescence, fresh PBS was gently injected into the subphase for an additional 30 min rotation at 35 °C. A 100-μl aliquot of 500 μl of the subphase provided the T2 fluorescence value. Fluorescence of 100 μl of PBS was subtracted as background signal. T0, T1, and T2 values facilitated calculation of the association (Ka) and dissociation (Kd) constants of Cy3 or Cy3-labeled peptides into and from the OAHFA or meibum layer, as described in the Results section.

Similarly, tryptophan fluorescence signals (excitation = 280 nm; detection = 325–500 nm) of N-94 or SDC1 “Pep30-50” peptide (“Ctrl pep”) in PBS were collected in a quartz cuvette (282 QS 1.000) at 35 °C in the cuvette chamber of the same SpectraMax M3 microplate reader at low photomultiplier tube power, 1 flash per read, and wavelength cutoff of 325 nm. Area under the emission spectra ≥325 nm was used as signals. T0, T1, and T2 fluorescence values were collected as described above for Cy3-labeled peptides but with the whole 500 μl sample. Fluorescence of 500 μl of PBS was subtracted as background signal. Only OAHFA films were used.

For cell culture studies, T0 and T2 subphase samples containing N-94 were filter-sterilized for inclusion in human corneal epithelial (HCE-T) cell viability experiments and MALDI mass spectrometry. HCE-T cells were validated by short-tandem repeat profiling. Cells were seeded overnight in 96-well plates at a density of 1.5 x 10^4^ cells/ml and then treated with interferon-γ (1000 U/ml; Sigma-Aldrich, St Louis MO) and tumor necrosis factor (100 ng/ml; Peprotech, Cranbury NJ) with or without equal molar amounts of T0 or T2-released N-94, or with C-95, every 2 days for 7 days. Viability was assessed by the alamarBlue assay (Thermo Fisher Scientific, Waltham MA). Quantity of T2 released N-94 was quantitated from fluorescence values fitted into the equation noted in the Results section with mass values obtained *via* the extinction coefficient. This value was confirmed by immunodot blot analysis *versus* an N-94 standard curve (Fig. S6*C*).

Rabbit pharmacokinetic studies were performed by Covance Laboratories Inc using Covance standard operating procedures in accordance with the Wisconsin Department of Health Services, Radiation Protection Section, as licensed to Covance, and in compliance with Animal Welfare Act Regulations (9 CFT 3). 44 μM stocks of N-94-Y and N-94/C-6-Y were radioiodinated by Perkin-Elmer (Shelton, CT) to a final specific activity of respectively 24.2 and 2.72 μCi/μg with initial radiopurity respectively 69.62 and 97.65%. At Covance Laboratories Inc (Madison, WI), 35 μl (3.3 μCi) of 1.3 μM ^125^I-N-94 in PBS repeated three times over 10 min or a single 35 μl (10 μCi) dose of 44 μM ^125^I-N-94/C-6 in 10 mM sodium citrate, 137 mM sodium chloride (pH 6.5) were topically added to each eye of respectively 12 and 14 female pigmented New Zealand White/New Zealand Red F1 cross rabbits (>3 months old, >2500 g each; Covance Research Products; Denver PA). 0.25, 0.5, 1, 3, 6, or 12 h after the first dose, tears were collected (30 s from inferior cul de sac) using Tear Flo Test strips (Accutome, Malvern PA) without prior anesthesia. Blood was also collected (0.5, 1, 3, 6, and 12 h postdose) *via* an auricular artery canula into tubes containing K_2_EDTA and centrifuged to obtain plasma. Later in the day, ^125^I-N-94/C-6 treated eyes were further treated with 35 μl (10 μCi) of 44 μM of ^125^I-N-94/C-6 twice daily for three (^125^I-N-94/C-6) additional days. The final treatment was on the morning of the fifth day after which tears were collected at 0.5, 1, 3, 6, 12, and 24 h postdose from two animals per time point who were then euthanized for collection of blood and ocular tissues. Eyes of a second group of 14 rabbits were treated twice daily with 35 μl of 4 μM ^125^I-N-94 (8 μCi) for 3 days with the final treatment on the morning of the fourth day after which tears were collected at 0.5, 1, 3, 6, 12, and 24 h postdose from two animals per time point who were then euthanized for collection of blood and ocular tissues. Radiopurity determined upon dosing by size-exclusion chromatography was 67.2% on Day 1 and 45.2% on Day 4 for ^125^I-N-94 and 98.8% on Day 1 and 97.9% on Day 5 for ^125^I-N-94/C-6. TCA precipitable radioactivity of formulated ^125^I-N-94 and ^125^I-N-94/C-6 respectively averaged 78% and 94% over the 4 and 5 days of dosing. TCA precipitable radioactivity of samples was assessed by scintillation counting and expressed per tissue wet weight.

### Mass spectrometry

Stability of 50 μM N-94 and N-94/C-6 in 10 μl of human basal tears for 4 h at 37 °C was assessed by MALDI TOF mass spectrometry (Bruker MicroFlex) by diluting the digest 1:20 with 0.1% trifluoracetic acid for 1:1 application in a sinapinic acid matrix in 50% acetonitrile and 0.1% trifluoracetic acid for ionization. Mass measurements were taken in linear, positive mode. To appreciate how C-terminal proteoforms may be processed, 50 μM N-64/C-31 was incubated at 35 °C for 48 h with 38 μl of basal normal tears without or with individual inhibitors (G Biosciences, St Louis MO) diluted from stock as AEBSF (0.2 or 1 mM), antipain (74 or 370 μM), bestatin (130 or 650 μM), chymostatin (100 or 500 M), EDTA (2.5 or 5 mM), leupeptin (10 or 50 μM), and pepstatin (1 or 5 μM) in a reaction volume of 50 μl buffered by 20 mM HEPES, 150 mM NaCl, 10 mM CaCl_2_ (pH 7.4), or instead with 5% DMSO in the same buffer as vehicle control. Samples were then processed as above after spiking in fresh 4 μM N-94/C-6 during the 0.1% TFA dilution for combination with sinapinic acid matrix.

## Statistical analyses

All experiments were performed at least three times, with the exception of some Brewster angle microscopy and ^125^I-N-94 and -N-94/C-6 pharmacokinetic studies. Data are reported as the mean ± SD with statistical approaches detailed in figure legends, as performed in Prism 8.4.3.

## Data availability

Source data for Figure 1, Figure 2, Figure 3, Figure 4, Figure 5 and Figures S1–S4 and S6 are provided in Data files, as indicated in each figure legend. Other supportive data of this study are available by request to the corresponding author.

## Conflict of interest

G. W. L. is cofounder and CSO, and MGO is CMO of TearSolutions, Inc. G. A. G., M. S., J. R., K. D. T., C. S., D. S. R., R. K. S., J. P. K., J. M. H., K. L. H., A. L., T. S., R. L. M. declare no conflict of interest.
