# Supplementary material for: Lacritin proteoforms prevent tear film collapse and maintain epithelial homeostasis
Source: J Biol Chem. 2020 Nov 21;296:100070. doi: 10.1074/jbc.RA120.015833 (PMC7948570; doi:10.1074/jbc.RA120.015833)
Supplement: Figures S1-S6 [file mmc10.pdf]

# SUPPORTING INFORMATION

## Lacritin proteoforms prevent tear film collapse and maintain epithelial homeostasis

Georgi A. Georgiev[1^*], Mohammad Sharifian Gh.[2^], Jeff Romano[2^], Karina L. Dias Teixeira[2], Craig Struble[3], Denise S. Ryan[4], Rose K. Sia[4], Jay P. Kitt[5], Joel M. Harris[5], Ku-Lung Hsu[6], Adam Libby[6], Marc G. Odrich[7], Tatiana Suárez[8], Robert L. McKown[9], and Gordon W. Laurie[2,7,10*]

[1]Institute for Bioengineering and Biosciences, Instituto Superior Técnico, Universidade de Lisboa, Lisbon, Portugal
[2]Department of Cell Biology, University of Virginia, Charlottesville VA, USA
[3]Covance Laboratories Inc, Madison WI, USA
[4]Warfighter Refractive Eye Surgery Program and Research Center at Fort Belvoir, Fort Belvoir VA, USA
[5]Department of Chemistry, University of Utah, Salt Lake City, UT
[6]Department of Chemistry, University of Virginia, Charlottesville VA, USA
[7]Department of Ophthalmology, University of Virginia, Charlottesville VA, USA
[8]Department of Research, Development and Innovation, FAES FARMA, Bizkaia, Spain
[9]Department of Integrated Science and Technology, James Madison University, Harrisonburg VA, USA
[10]Department of Biomedical Engineering, University of Virginia, Charlottesville VA, USA

*Corresponding author: Gordon Laurie

Email: glaurie@virginia.edu

**Running title:** Lacritin Tear Stabilization

**Keywords:** lacritin, proteoform, eye, tears, OAHFA, protein-lipid interaction, elastic modulus, Raman spectroscopy, proteolysis, epithelium

**Supporting Figures S1 - S6, plus Data Files for Figures 1 - 5 and Supporting Figures S1 - S4, and S6.**

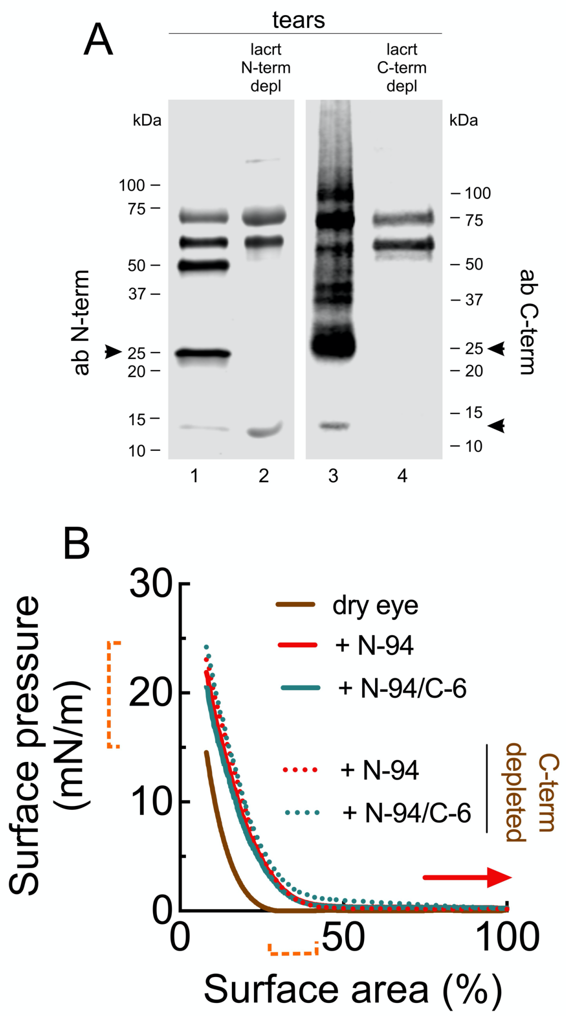


**Figure S1: Immunodepletion of lacritin monomer and C-terminal proteoform; and N-94 or N-94/C-6 restoration of dry eye tear stability under expansion. A.** Ab N-term (left) and ab C-term (right) lacritin Western blots of human basal tears, or human basal tears subjected to immunodepletion respectively with ab N-term (1F5 mab) or ab C-term. Lacritin in tears appears for the most part as a monomer (~25 kDa), and larger multimers (Velez et al, '13). The <15 kDa band respectively represents N- and C-terminal proteoforms. **B.** Expansion profile of dry eye tears without or with added N-94 or N-94/C-6; or C-terminal depleted normal tears with added N-94 or N-94/C-6 (expansion profile of C-terminal depleted tears is shown in Fig. 1E). Y-axis bracket indicates the comparative difference in highest surface pressure attained under compression by N-94 or N-94/C-6 supplemented dry eye or lacritin depleted normal tears (upper portion) versus dry eye or lacritin depleted normal tears (lower portion). X-axis bracket compares the set down point of dry eye (left) versus supplemented tears (right). Each isotherm represents the mean of triplicate experiments with individual experiments representing over 1200 data points. Data for **B** in **Data files Figure 1 (expansion isocycling)**.

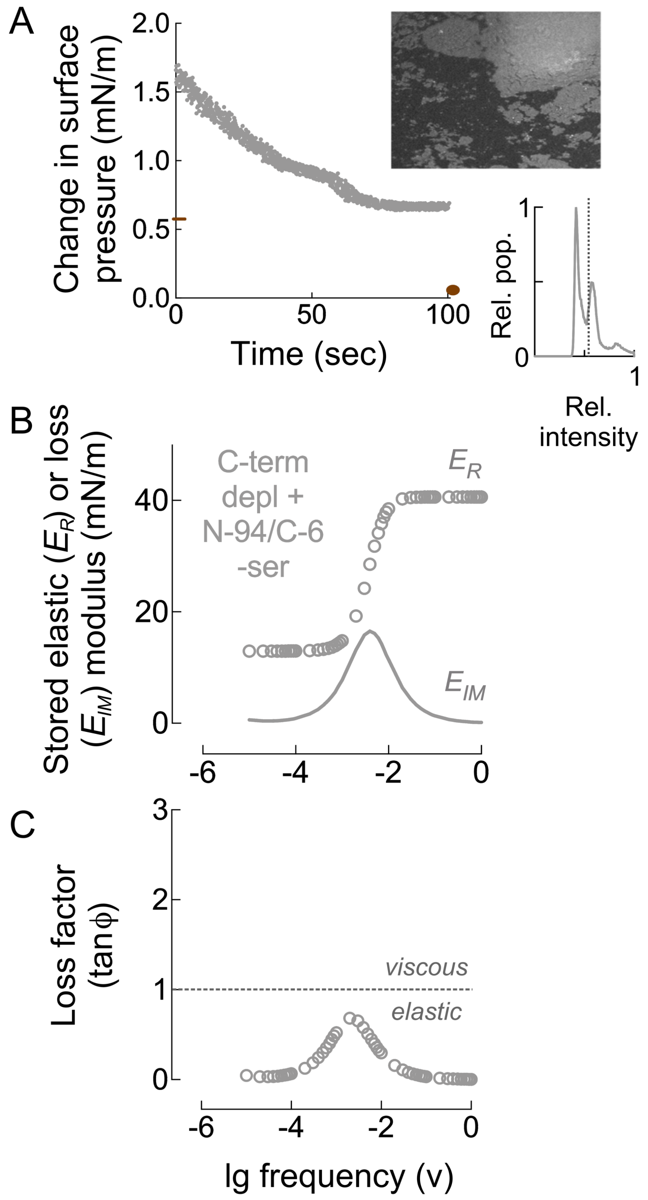


**Figure S2: N-94/C-6-ser restores normal viscoelasticity to lacritin depleted tears. A.** Time-dependent relaxation of surface tension after pre-equilibrated anti-C-term lacritin-depleted tears with 6 μM N-94/C-6-ser were subjected to a sudden step compression of less than 5% of the prior surface area. Brown line on y-axis and dot on X-axis indicates values for anti-C-term lacritin-depleted tears alone from Figure 2A. Shown are individual replicates for each experiment performed in triplicate (1260 data points). *Insets,* Brewster angle microscopy of anti-C-term lacritin-depleted tears with 6 μM N-94/C-6-ser, and ImageJ analysis (vertical dashed line indicates Relative Intensity of the maximal Relative Population in normal tears). Representative of triplicate experiments. **B.** Fourier transform of the relaxation data as the stored elastic ($E_R$) or loss ($E_{IM}$) moduli, or **C.**, loss factor (tan ϕ) - both as a function of the log frequency. Data for **A, B, C** in **Data files Supporting Figure S2**.

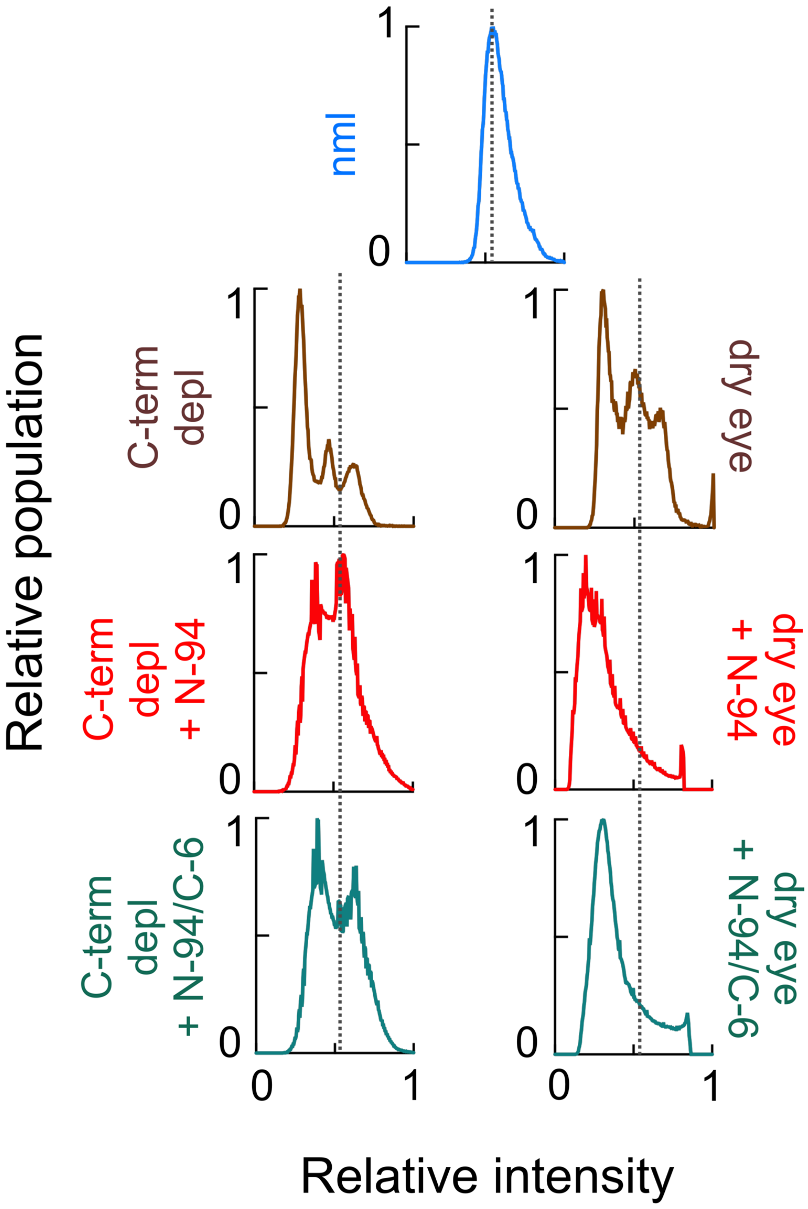


**Figure S3: ImageJ analysis of Brewster angle microscopy (Fig. 2H) reflects morphological similarities and differences respectively for normal (nml) tears versus supplemented anti-C-term lacritin-depleted or dry eye tears.** Vertical dashed line indicates Relative Intensity of the maximal Relative Population in normal tears. Representative of triplicate experiments. Data in **Data files Supporting Figure S3.**

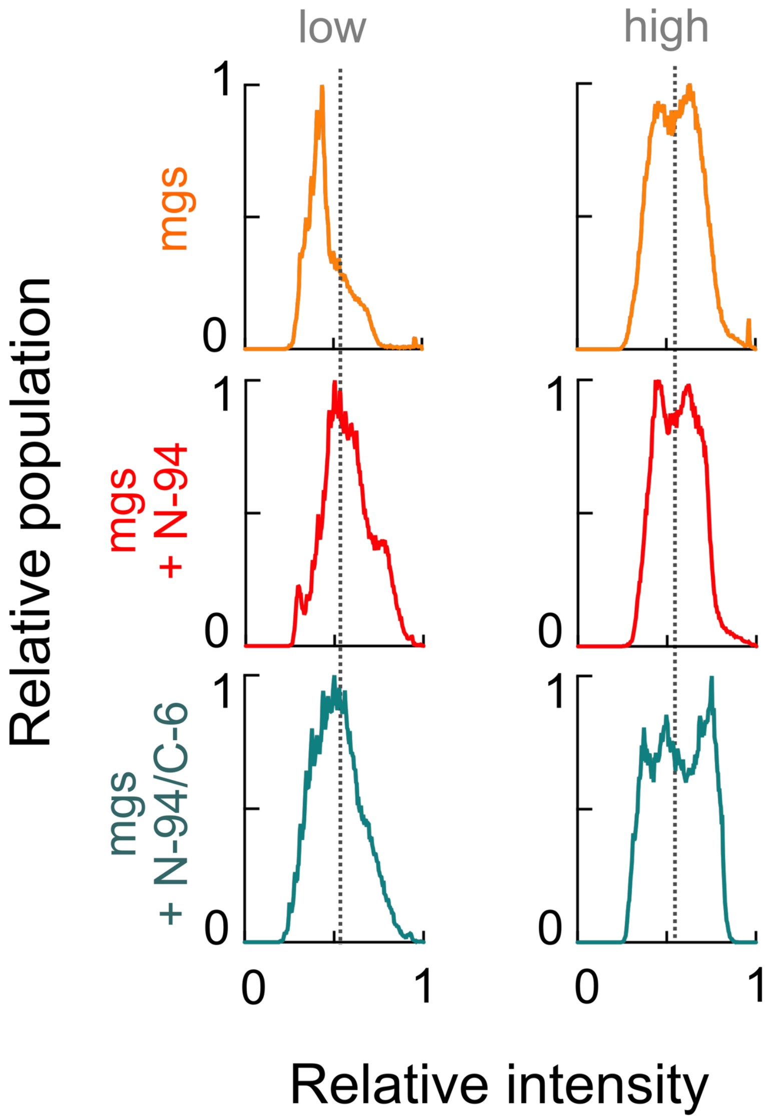


**Figure S4: ImageJ analysis of Brewster angle microscopy (Fig. 3E) reflects morphological similarities and differences for meibomian gland secretion (mgs) at low or high pressure without or with supplementation.** Vertical dashed line indicates Relative Intensity of the maximal Relative Population in normal tears. Representative of triplicate experiments. Data in **Data files Supporting Figure S4.**

$$\Pi = \left(a + \frac{(b \cdot k_1 - c \cdot k_2)}{(k_2 - k_1)} \cdot \exp(-k_1 \cdot t)\right)$$

$$+ \frac{k_1}{(k_2 - k_1)} \cdot (c - b) \cdot \exp(-k_2 \cdot t) + c.$$

**Π**, surface pressure

**k1**, first order docking rate constant

**k2**, first order incorporation rate constant

**a**, **b**, **c**, parameters describing the respective initial, intermediate and final states of overall reaction

| **Meib gl secret** | **$k_1$, $s^{-1}$** | **$k_2$, $s^{-1}$** | **a** | **b** | **c** |
|---|---|---|---|---|---|
| + N-94 | 2.678e-02 | 3.434e-04 | 1.447e+01 | 6.675 | 1.426e+01 |
| + N-94/C-6 | 2.139e-02 | 2,411e-03 | 1.544e+01 | 1.065e+01 | 1.333e+01 |

**Figure S5: N-94 and N-94/C-6 penetration kinetics into films of meibomian gland secretion.** Suspected film perturbation by rapid N-94 or N-94/C-6 docking increases surface pressure that further increases with slower incorporation.

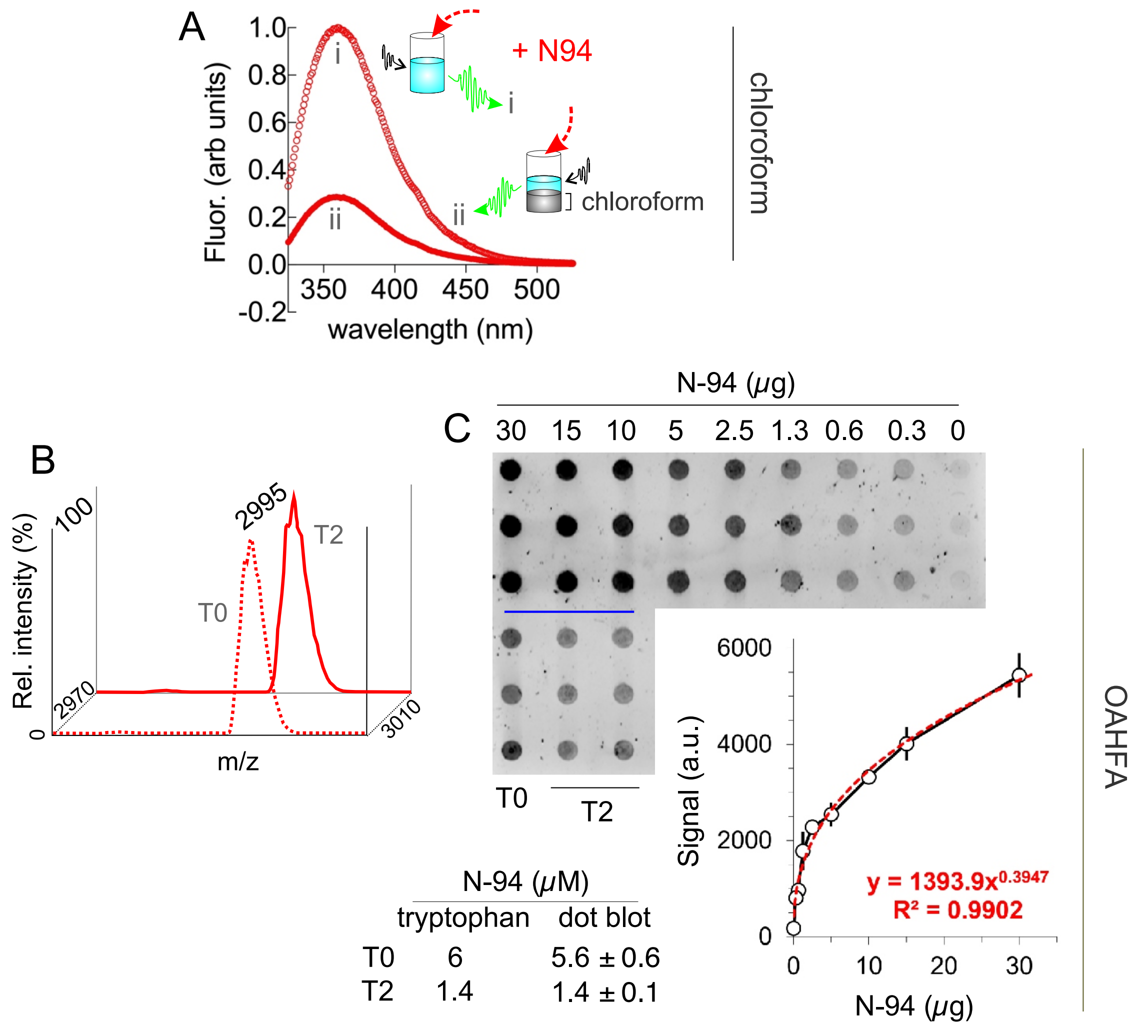


**Figure S6: Chloroform quenches N-94's penultimate tryptophan; and N-94 released from OAHFA is intact.** **A.** Tryptophan emission spectra of N-94 in the absence (i) or presence (ii) of chloroform (respectively representative of three replicated experiments, and n = 3). **B.** MALDI TOF mass spectrometry of equimolar amounts (1.4 µM) of N-94 at T0, or after release from OAHFA (T2; both T0 and T2 as per Fig. 4C scheme). Mass measurements were taken in linear, positive mode (n = 1). **C.** Ab-C-term detection of decreasing dotted amounts of N-94 vs T0 and T2 N-94 for determination of the latter, versus amounts calculated by fluorescence (n = 1). Data for **A** in **Data files Supporting Figure S6**.
